# Chemistry of Phosphorylated Formaldehyde Derivatives. Part I

**DOI:** 10.3390/molecules190912949

**Published:** 2014-08-25

**Authors:** Vasily P. Morgalyuk

**Affiliations:** Laboratory of Organophosphorus Compounds, Nesmeyanov Institute of Organoelement Compounds, Russian Academy of Sciences, Vavilova str., 28, Moscow 119991, Russia; E-Mail: morgaliuk@mail.ru; Tel.: +7-499-135-92-50; Fax: +7-495-135-65-49

**Keywords:** phosphorylated acetals, -thioacetals, -aminonitriles, -aminomethylphosphinoyl compounds, -chloro(or bromo)aminals, -gem-dioles, -ketene acetale, H-phosphinate

## Abstract

The underinvestigated derivatives of unstable phosphorylated formaldehyde acetals and some of the structurally related compounds, such as thioacetals, aminonitriles, aminomethylphosphinoyl compounds, are considered. Separately considered are halogen aminals of phosphorylated formaldehyde, acetals of phosphorylated formaldehyde of H-phosphinate-type and a phosphorylated gem-diol of formaldehyde. Synthetic methods, chemical properties and examples of practical applications are given.

## 1. Introduction

Among organophosphorus compounds, α-phosphorylated carbonyl compounds stand out by the capacity of cleavage of phosphorus–carbon bond under mild conditions when reacted with nucleophiles [1,2,3,4,5]. The cleavage of phosphorus–carbon bond may proceed spontaneously as well. At the same time, α-oxoalkylphosphinoyl compounds also retain properties inherent in carbonyl compounds, for example, they undergo cross aldol condensation.

**Figure 1:**
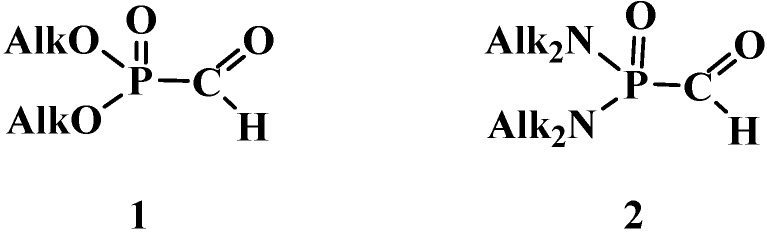
Structures of dialkyl formylphosphonates (**1**) and *N,N,N',N'*-tetraalkyl formylphosphondiamides (**2**).

The least stable compounds among them are phosphorylated formaldehyde derivatives, dialkyl formylphosphonates (**1**) [6,7,8,9,10,11] and *N,N,N',N'*-tetraalkyl formylphosphondiamides (**2**) [12] (Figure 1), whose existence was even disputed in the first half of the 1970s [13,14].

**Scheme 1:**
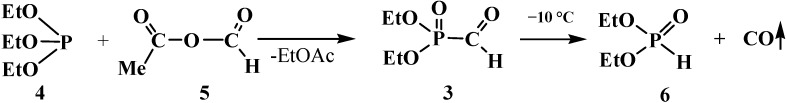
Syntheses and destruction of diethyl formylphosphonate (**3**).

However, the first synthesis of **1** by reaction of sodium derivatives of dialkyl phosphites with acetic formic anhydride was described in 1974 [15]. Compounds **1** were shown to be unstable and prone to spontaneous degradation [9,10,16,17]; thus, diethyl formylphosphonate (**3**) prepared by the reaction of triethyl phosphite (**4**) with acetic formic anhydride (**5**) at low temperature begins to undergo decarbonylation even at −10 °C [9,10] to give diethyl phosphite (**6**) (Scheme 1).

**Scheme 2:**
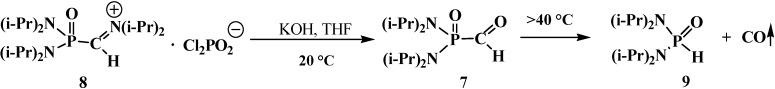
Syntheses and destruction of *N,N,N',N'*-tetraisopropyl formylphosphondiamide (**7**).

*N,N,N',N'*-Tetraisopropylformylphosphondiamide (**7**) obtained by saponification of *N,N,N′,N′*- tetraisopropyl[(*N'',N''*-diisopropylamino)methylydeniminium]phosphondiamide dichlorophosphate (**8**) with potassium hydroxide in tetrahydrofuran at 20 °C proved to be slightly more stable; it undergoes decarbonylation only above 40 °C [12] to give *N,N,N',N'*-tetraisopropylphosphonicdiamide (**9**) (Scheme 2).

**Scheme 3:**
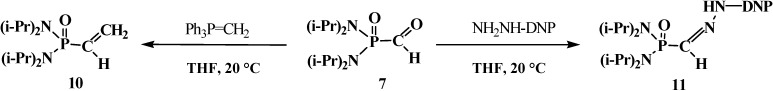
Reactions *N,N,N',N'*-tetraisopropylformylphosphondiamide (**7**) as an aldehyde (DNP means a 2,4-dinitrophenyl moiety).

The stability of the formylphosphondiamide **7** allowed the study some of its chemical properties. It was shown that in its reactions with methylene(triphenyl)phosphorane (Ph_3_P=CH_2_) and 2,4-dinitrophenylhydrazine (NH_2_NH-DNP) **7** behaves as a typical aldehyde, yielding the corresponding α-phosphorylated olefin **10** and hydrazone **11** [12] (Scheme 3).

**Figure 2:**
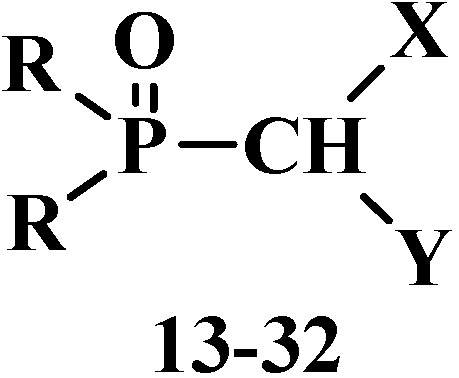
Molecular structure of phosphorylated formaldehyde acetals (**13**) and related compounds (**14**–**32**), where R = OAlk, Alk_2_N, Ph; X, Y are the combinations of OAlk, Alk_2_N, CN, (AlkO)_2_P(O), S(Alk, Ar), S(O)(Alk, Ar), N(H)C(O)Alk, C(O)Ar, N(H)S(O)_2_Alk, S(O)_2_Ar, Hal, Me_3_Si [18,19,20,21,22,23,24,25,26,27,28,29,30,31,32,33,34,35,36,37,38,39,40,41,42,43,44,45,46,47,48,49,50,51,52,53,54,55,56].

However, many authors showed that formylphosphinoyl compounds are also stable in the form of formylphosphonic acid (**12**) [12,14,15], its disodium salt [15], aldehyde group derivatives such as acetals **13** [18,19,20] and structurally related compounds. These compounds are thioacetals **14** [21,22,23], aminonitriles **15** [24,25,26], diphosphinoyl (*N,N*-dialkylaminomethyl)methanes **16** [27,28,29], chloro(or bromo)aminals **17** [30,31,32,33,34], mixed S,O-thioacetals **18** [35,36,37], aminals **19** [30,38], aminoacetals **20** [31,32,38,39], aminothioacetals **21** [32], chloroacetals **22** [40,41], chloro- (or bromo-) thioacetals **23** [40,42,43], α-chlorosulfinyl derivatives **24** [44,45], α-alkoxynitriles **25** [46,47,48], α-thionitriles **26** [46], α-dihalo derivatives **27** [49,50,51], α-alkoxydiphosphoryl compounds **28** [41], α-mercaptodiphosphoryl compounds **29** [40,52,53], α-alkoxysilyl derivatives **30** [54], α-aminosilyl derivatives **31** [29,55], or α-mercaptosilyl derivatives **32** [56] (Figure 2):

Among these compounds, phosphorylated acetals **13**, thioacetals **14**, α-dimethylaminonitriles **15**, aminodiphosphinoyl compounds **16** and chloro- (or bromo-) aminals **17** are used in contemporary organic synthesis. Nonetheless, the phosphorylated acetals of formaldehyde and structurally related compounds remain poorly studied types of organophosphorus compounds until now. The chemical properties of this type of compounds were most studied on an example of dialkyl(dialkoxymethyl)phosphonates **33**. The properties of other compounds **18**–**32** have been studied in much less detail, and the majority of them are examined in one-sided manner, only as precursors for the synthesis of ketene acetals and similar compounds by the Horner reaction.

**Figure 3:**
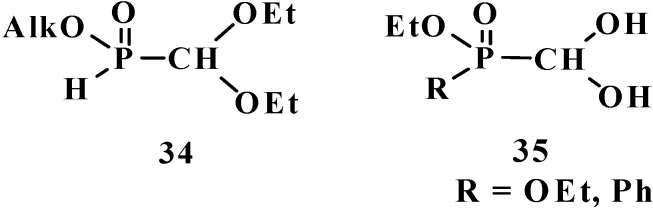
Molecular structure of alkyl (diethoxymethyl)phosphinate **34** and phosphorylated formaldehyde hydrates **35**.

To date, it is known that—except for formylphosphonic acid (**12**), phosphorylated acetals **13**, and similar compounds **14**–**32**—the phosphorylated formaldehyde derivatives are stable in the form of H-phosphinate acetals: alkyl (diethoxymethyl)phosphinate **34** [57,58,59], and geminal dioles, phosphorylated formaldehyde hydrates (hydrates of phosphorylated formaldehyde) **35** [60,61,62] (Figure 3).

**Figure 4:**
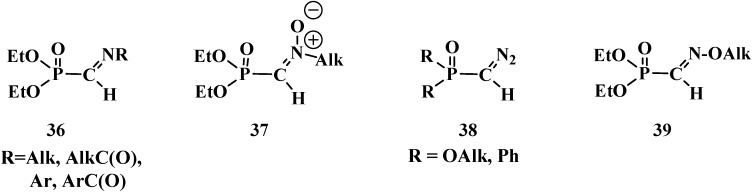
Molecular structures of formylphosphonates of *N*-substituted imines **36**, N-alkylnitrones **37**, phosphorylated diazomethane **38** and alkylated oximes **39**.

Noteworthy are nitrogen-containing analogs of phosphorylated formaldehyde: *N*-substituted imines (**36**) [16,63,64], *N*-alkylnitrones **37** [8,65,66], phosphorylated diazomethane (**38** [67,68,69] and O-alkylated oximes of diethyl formylphosphonates **39** [7,70], used in contemporary organic synthesis (Figure 4).

However, the properties of compounds **36**–**39** differ significantly from those of compounds **13**–**32**. Therefore, compounds **36**–**39** will be considered in a separate publication.

## 2. Chemistry of Phosphorylated Formaldehyde Derivatives

### 2.1. Syntheses and Chemical Properties of Formylphosphonic Acid (**12**)

**Scheme 4:**
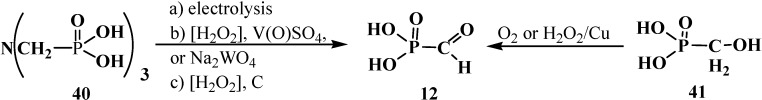
Possible routes of syntheses of formylphosphonic acid (**12**).

Formylphosphonic acid (**12**), which shows distinct antiviral activity [71] was obtained for the first time in 1974 [14] as a byproduct of the electrolysis of an aqueous solution of nitrilotrimethylphosphonic acid (**40**) (Scheme 4). Later it was shown that **12** might be also obtained in high yields by the catalytic oxidation of nitrilotrimethylphosphonic acid (**40**) with hydrogen peroxide in the presence of vanadyl sulfate or potassium tungstate (88%–93% yield) [17] or activated carbon (up to 82% yield) [72]. Compound **12** can be also prepared by the oxidation of hydroxymethylphosphonic acid (**41**) in aqueous solution with air oxygen or hydrogen peroxide in the presence of Raney copper in a yield up to 65% [73] (Scheme 4).

**Scheme 5:**
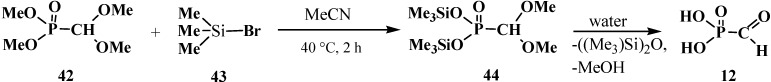
Synthesis of formylphosphonic acid (**12**) from dimethyl (dimethoxymethyl)phosphonate (**42**).

In a third method of synthesis, **12** was obtained via a two-stage synthesis starting from dimethyl (dimethoxymethyl)phosphonate (**42**), which was converted by treatment with bromotrimethylsilane (**43**) into bis(trimethylsilyl)(dimethoxymethyl)phosphonate (**44**). Hydrolysis of the latter resulted in formylphosphonic acid (**12**) [74] (Scheme 5). See also Section 3.2.—Synthesis of formacetalphosphonic acids.

**Scheme 6:**
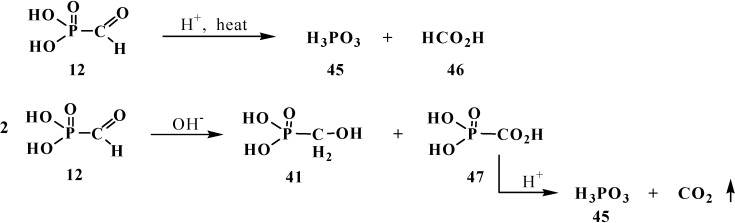
Degradation of formylphosphonic acid (**12**) to form phosphoric **45** and formic acids **46** and disproportionation of **12** in aqueous solutions.

However, the chemical properties of formylphosphonic acid (**12**) have been poorly studied until now. It is known that heating **12** in acidic medium leads to the cleavage of the P–C bond to form phosphoric and formic acids **45** and **46**. In the presence of bases **12** undergoes disproportionation (Cannizzaro reaction) to give hydroxymethylphosphonic **41** and carboxyphosphonic acids **47**. Acid **47** undergoes rapid decarboxylation on acidification of the reaction medium [14] (Scheme 6).

### 2.2. Chemistry of Phosphorylated Formaldehyde Acetals **13**

Chemistry of phosphorylated formaldehyde acetals **13** started as a chemistry of dialkyl (dialkoxymethyl)phosphonates **33** due to their more ready availability as compared with the analogs—*N,N,N',N'*-tetraalkyl(dialkoxymethyl)phosphondiamides **48**, dialkyl (dialkoxymethyl)-phosphine oxides **49** or diphenyl(dialkoxymethyl)phosphine oxide **50**. Many types of acetals are known to date, but their chemical properties are still insufficiently studied, although they are more studied than the other derivatives of phosphorylated formaldehyde.

#### 2.2.1. Methods of Synthesis of Phosphorylated Formaldehyde Acetals **13**

**Scheme 7:**
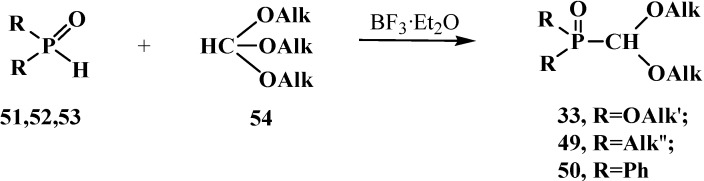
Synthesis of phosphorylated formaldehyde acetals **33**, **49**, **50** from dialkyl phosphites **51** or *sec*-phosphine oxides **52**, **53** by means of orthoformate esters **54**.

First phosphorylated formaldehyde acetals **13** were obtained by the reaction of hydrophosphinoyl compounds **51**–**53** with orthoformate esters **54** on heating. The reaction with dialkyl phosphites **51** [19,75] is conducted by heating to 182 °C [76,77] or at 60 °C in the presence of BF_3_·Et_2_O as catalyst (without the catalyst the yields decrease from 69%–90% to 25% [75]). In the case of *sec*-phosphine oxides—dialkylphosphine oxides **52** [77] and diphenylphosphine oxide **53** [77,78], the reaction proceeds at 100 °C [19,77]. This method provides high yields of compounds **33**, **49** and **50** (Scheme 7).

**Scheme 8:**
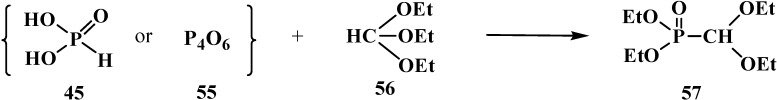
Synthesis of diethyl (diethoxymethyl)phosphonate (**57**) from phosphoric acid (**45**) or phosphorous anhydride (**55**).

A variant of this method consists in the reaction of phosphoric acid (H_3_PO_3_, **45**) or phosphoric anhydride (**55**) with triethyl orthoformate (**56**) in 1:3 ratio that results in diethyl (diethoxymethyl)phosphonate (**57**) with minimal effort [79] (Scheme 8).

**Scheme 9:**
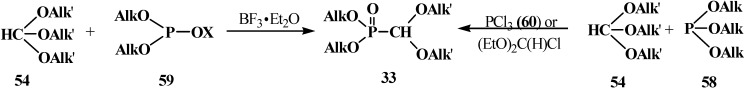
Synthesis of dialkyl (dialkoxymethyl)phosphonates **33** from orthoformate esters **54**, trialkyl phosphites **58** and their analogs (AlkO)_2_POX **59**.

Compounds **33** can be also prepared by the reaction of trialkyl phosphites **58** with orthoformate esters **54**. It was shown that phosphites **58** do not react directly with **54** even on heating [19]. However, as in the case of dialkyl phosphites **51**, orthoformates **54** react with trialkyl phosphites **58** and their analogs (AlkO)_2_POX **59**, where X = Me_3_Si, (AlkO)_2_P, (AlkO_2_)P(O) on heating in the presence of catalytic amounts of boron trifluoride etherate BF_3_·Et_2_O [19]. The reaction of **58** with orthoformate esters **54** is also possible in the presence of phosphorus trichloride (PCl_3_, **60**) or diethoxychloromethane (EtO)_2_C(H)Cl [80,81] (Scheme 9).

**Scheme 10:**
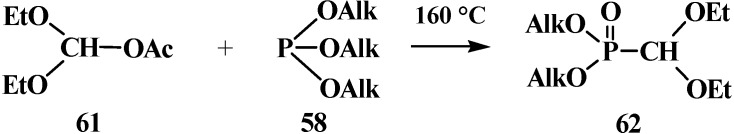
Synthesis of dialkyl (diethoxymethyl)phosphonates **62** from orthoformate esters **54** and acetoxy(diethoxy)methane (**61**).

It was reported that the reaction of trialkyl phosphites **58** with acetoxy(diethoxy)methane (**61**), which is more reactive derivative than orthoformate esters **54** [19,82], results in dialkyl (diethoxymethyl)phosphonates **62** (Scheme 10).

**Scheme 11:**
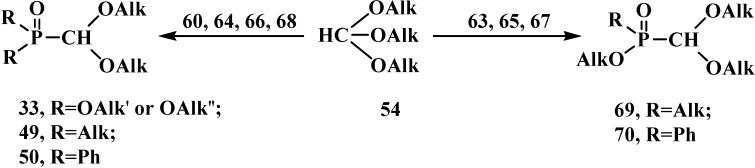
Syntheses of phosphorylated formaldehyde acetals **33**, **49**, **50**, **69**, **70** from phosphorus trichloride derivatives **63**–**68** with orthoformate esters **54**. See the text above.

The most general method of synthesis of phosphorylated formaldehyde acetals **13** is the reaction of phosphorus trichloride derivatives **60** with orthoformate esters **54**. Excess of **54** reacts on heating with **60** [79,81,83,84,85] and mono- and dialkyl AlkPCl_2_**63**, Alk_2_PCl **64**, alkoxy (AlkO)PCl_2_**65**, (AlkO)_2_PCl **66**, and phenyl PhPCl_2_**67**, Ph_2_PCl **68** [80,84,86,87,88,89] derivatives of **60** (Scheme 11), to form symmetrical and unsymmetrical acetals **33**, **49**, **50** and **69**, **70**, respectively. It was noted that the reaction of 2-chloro-1,2,3-dioxaphospholanes **71** with **54** leads to the opening of the dioxaphospholane ring to form ethyl (β-chloroethyl) ([1,3]-dioxolan-2-yl)phosphonates **72** [90].

**Scheme 12:**
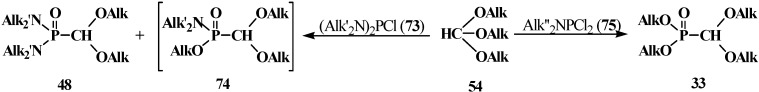
Interaction of *N,N,N',N'*-tetraalkyl(chloro)phosphindiamides **73** and *N,N*-dialkyl(dichloro)phosphinamides **75** with orthoformates **54**.

The reaction of *N,N,N',N'*-tetraalkyl(chloro)phosphindiamides **73** with orthoformates **54** proceeds with a partial exchange of dialkylamino groups at phosphorus atom of **73** to give both *N,N,N',N'*-tetraalkyl (dialkoxymethyl)phosphondiamides **48** and mixed phosphonamidates **74** [19,91,92]. In the case of *N,N*-dialkyl(dichloro)phosphinamides **75**, a total exchange of dialkylamino groups at the phosphorus atom for alkoxy groups takes place to yield dialkyl (dialkoxymethyl)phosphonates **33** [85] (Scheme 12).

**Scheme 13:**
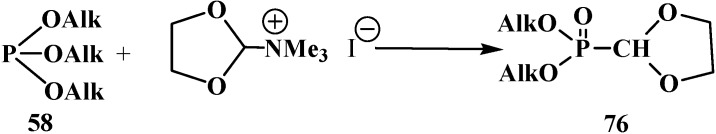
Synthesis of cyclic acetals **76** by interaction of trialkyl phosphites **58** with the quaternary ammonium salts of dimethylformamide acetals.

Quaternary ammonium salts of dimethylformamide acetals can be used instead of orthoformates **54** [18,93]. The method only allows preparation of cyclic phosphorylated formaldehyde dialkyl acetals **76** since the quaternary ammonium salts of dialkylformamide acetals of linear structure are unstable and undergo fast degradation [93] (Scheme 13).

Nonetheless, linear compounds **33** can be obtained by this method in low yield (15%–25%) using a one-pot method from methyl iodide, dialkylformamide acetals **77**, and trialkyl phosphites **58** [18].

**Scheme 14:**
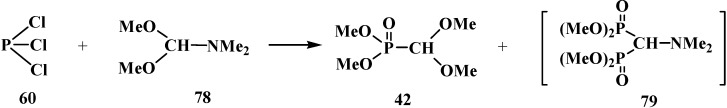
Syntheses of dimethyl (dimethoxymethyl)phosphonate (**42**) from phosphorus trichloride (**60**) and dimethylformamide dimethylacetal (**78**).

The reaction of phosphorus trichloride (**60**) with dimethylformamide dimethylacetal (**78**) also leads to the formation of dimethyl (dimethoxymethyl)phosphonate (**42**) (along with a certain amount of tetramethyl (*N,N*-dimethylaminomethyl)diphosphonate (**79**)) [83] (Scheme 14).

**Scheme 15:**
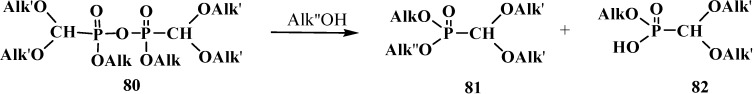
Preparation of acetals **81** with different alkoxy substituents at phosphorus atom by means of alcoholysis of tetraalkyl bis(dialkoxymethyl)pyrophosphonates **80**.

Dialkyl acetals **33** can be also prepared by the reaction of alcohols with tetraalkyl bis(dialkoxymethyl)pyrophosphonates **80**. The method provides a possibility to obtain formaldehyde dialkyl acetals with different alkoxy substituents at phosphorus atom **81** [89] that are difficult to prepare from trivalent phosphorus derivatives and orthoformates **54** or their derivatives because of competitive exchange of substituents at phosphorus atom [19,89] (Scheme 15). Alkyl (dialkoxymethyl)phosphonic acid **82** is also formed at the same time. See also Scheme 22 and Scheme 23.

**Scheme 16:**
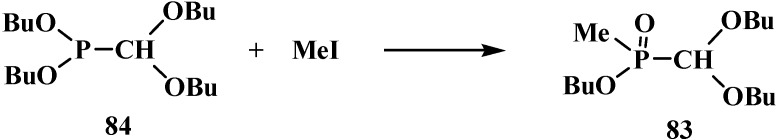
Example synthesis of acetals with different substitutients at phosphorus atom by the Arbuzov reaction. See also Scheme 11, Scheme 15, Scheme 17, Scheme 21, Scheme 22, Scheme 23, Scheme 111, Scheme 112, Scheme 113 and Scheme 114.

Alkyl alkyl(dialkoxymethyl)phosphinates **83** unsymmetrically substituted at the phosphorus atom were synthesized by the Arbuzov reaction of dialkyl (dialkoxymethyl)phosphonites **84** with alkyl iodides [82,84], for example see Scheme 16.

**Scheme 17:**
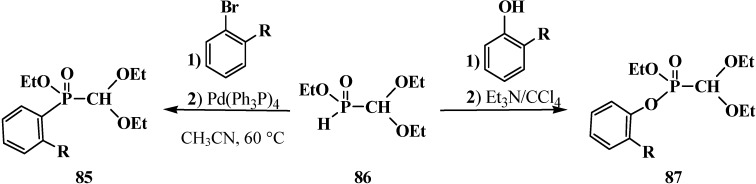
Synthesis of ethyl aryl(diethoxymethyl)phosphinates **85** and ethyl aryl (diethoxymethyl)phosphonates **87** from ethyl (diethoxymethyl)phosphinate (**86**), R are Alk or Hal.

The catalytic synthesis of ethyl aryl(diethoxymethyl)phosphinates **85** by the reaction of ethyl (diethoxymethyl)phosphinate (**86**) with *ortho*-substituted aryl bromides in the presence of tetrakis(triphenylphosphine)palladium(0) Pd(Ph_3_P)_4_ in 75%–90% yields was reported in 1995 [94]. The Todd-Atherton reaction of **86** with *ortho*-substituted phenols in the presence of triethylamine at 0–23 °C leads to ethyl aryl (diethoxymethyl)phosphonates **87** [94] (Scheme 17).

**Scheme 18:**
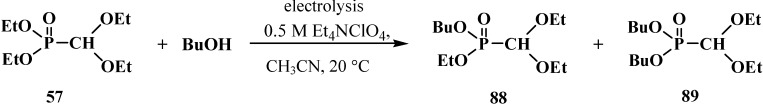
Synthesis of acetales **88** and **89** by transesterification under the conditions of electrolysis.

The attempted preparations of novel acetales **33** by the transesterification of phosphorus ester groups failed because they gave rise to intractable mixtures of compounds [95]. Ethoxyphosphoryl groups in acetal **57** were replaced by butoxyphosphoryl groups only under cathode electrolysis conditions. As a result, butyl ethyl (diethoxymethyl)phosphonate (**88**) and dibutyl (diethoxymethyl)phosphonate (**89**) were obtained in low yields—11% and 5%, respectively [96] (Scheme 18).

**Scheme 19:**
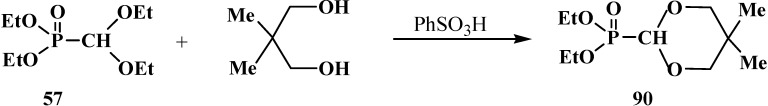
Preparation of cyclic diethyl (5-dimethyl-[1,3]-dioxan-2-yl)phosphonates **90** by the transesterification under acid catalysis.

However, the heating of **57** with 1,3-dimethylpropanediols in the presence of a catalytic amount of benzenesulfonic acid (PhSO_3_H) results in the replacement of ethoxy groups of the acetal fragment to give cyclic diethyl (5-dimethyl-[1,3]-dioxan-2-yl)phosphonates **90** [97], for example, see Scheme 19.

#### 2.2.2. Chemical Properties of Phosphorylated Formaldehyde Acetals **13**

The chemistry of phosphorylated formaldehyde acetals **13** was initially developed for the most part as a chemistry of available dialkyl (dialkoxymethyl)phosphonates **33**. Therefore the properties of acetals as a separate type of organophosphorus compounds were studied mainly by the examples of compounds **33** whose reactivity is affected by the presence of both phosphorus ester and acetal groups.

**Scheme 20:**
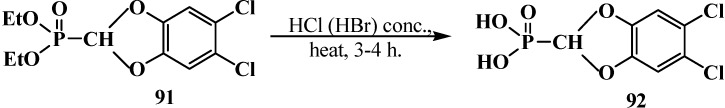
Acid hydrolysis of diethyl (5,6-dichloro-1,3-benzodioxomethyl)phosphonate (**91**).

Hydrolysis of acetals **33** was studied by the example of compound **57** and a compound with aromatic substituents in the acetal group, diethyl (5,6-dichloro-1,3-benzodioxomethyl)phosphonate (**91**). However, the attempted acid hydrolysis of acetal **57** on heating lead to the cleavage of phosphorus–carbon bond [77,95,98]. Compound **91** underwent acid hydrolysis on heating to give (5,6-dichloro-1,3-benzodioxomethyl)phosphonic acid (**92**) (Scheme 20). See also the section “Cleavage of Phosphorus–Carbon Bond under the Action of Acids and Acidic Reagents”, Scheme 54.

**Scheme 21:**
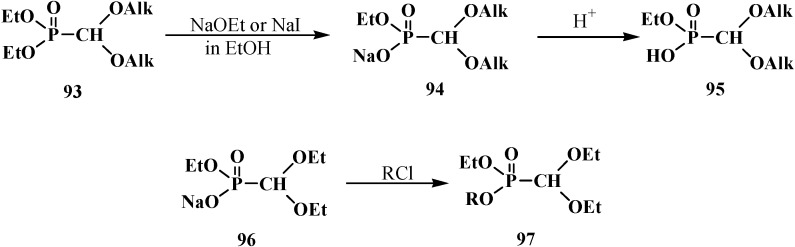
Acetals **93** dealkylation at their interaction with sodium ethoxide or sodium iodide. Synthesis of acetals **97** with different substituents at phosphorus atom, where R = Alk, Ac, Me_3_Si, MeOCH_2_, ArOCH_2_.

The heating of a solution of diethyl (dialkoxymethyl)phosphonate **93** in absolute ethanol with sodium ethoxide (NaOEt) leads to dealkylation of one of the ethoxy groups by phosphorus atom to form ethyl sodium (dialkoxymethyl)phosphonate **94**, which produces the free acid **95** on acidification [95]. Heating of **93** with sodium iodide NaI leads to the same result [93]. The reaction of ethyl sodium (diethoxymethyl)phosphonate (**96**) with electrophilic reagents brings about the formation of phosphonates **97** with different substituents at the phosphorus atom [95] (Scheme 21).

**Scheme 22:**
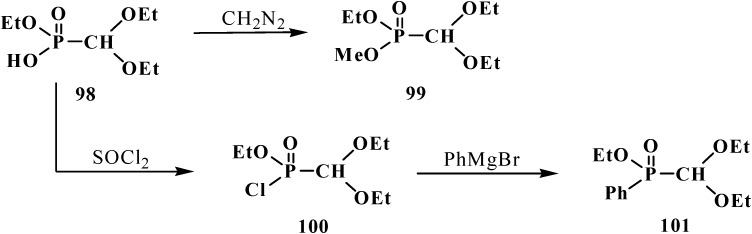
Ethyl (diethoxymethyl)phosphonic acid (**98**) transformations.

Acids **95** show typical properties of hydroxy compounds. Using as example ethyl (diethoxymethyl)phosphonic acid (**98**) it is shown that they react with diazomethane and thionyl chloride. The reaction products are ethyl methyl (diethoxymethyl)phosphonate (**99**) and ethyl (diethoxymethyl)phosphonic chloride (**100**) [95], which reacts with phenylmagnesium bromide in tetrahydrofuran to produce ethyl phenyl(diethoxymethyl)phosphinate (**101**) (Scheme 22).

**Scheme 23:**
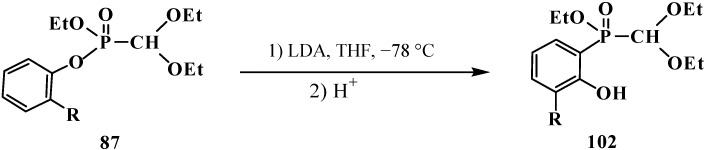
Rearrangement of compounds **87**, R = Alk, Hal.

Unsymmetrical ethyl aryl (diethoxymethyl)phosphonates **87** containing aryloxy substituents at the phosphorus atom [94] undergo rearrangement in the presence of equimolar amount of lithium diisopropylamide LDA in tetrahydrofuran at −70 °C to yield ethyl (2-hydroxyaryl)- (diethoxymethyl)phosphinates **102** (Scheme 23).

**Scheme 24:**
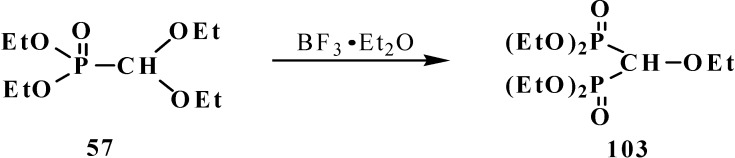
Formation of tetraethyl (ethoxymethyl)diphosphonate (**103**) from acetal **57** under a catalysis by BF_3_·Et_2_O.

Heating of diethyl (diethoxymethyl)phosphonate (**57**) with a catalytic amount of BF_3_·Et_2_O gives rise to formation of tetraethyl (ethoxymethyl)diphosphonate (**103**) in a 14% yield [19] (Scheme 24).

The acetal group of compounds **13** is rather stable to the action of co-reactants. Nonetheless, a series of transformations of phosphorylated formaldehyde acetals (**33**) that involves dialkoxyacetal group is described.

**Scheme 25:**
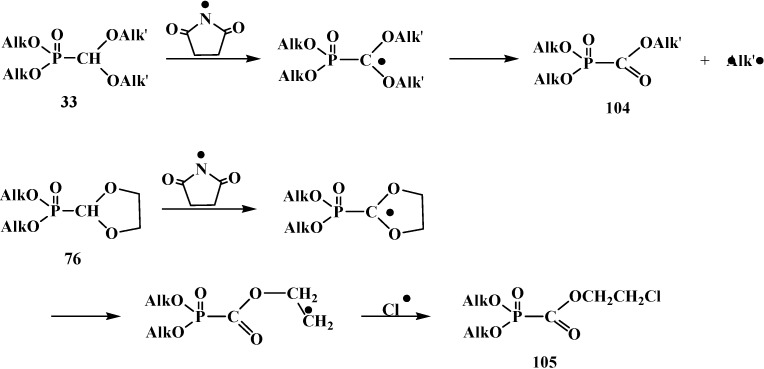
Interaction of compounds **33** and **76** with N-bromosuccinimide or N-chlorosuccinimide.

The halogenation of compounds **33** with *N*-bromosuccinimide leads to alcoxycarbonylphosphonates **104**. Five-membered cyclic acetals **76** by halogenation with *N*-chlorosuccinimide and azodiisobutyro-nitrile mix produce β-haloethoxycarbonylphosphonates **105** [99]. Reactions proceed via a radical mechanism (Scheme 25).

**Scheme 26:**
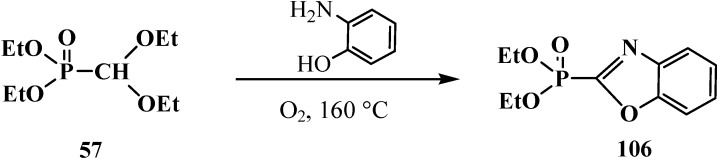
The reaction of diethyl (diethoxymethyl)phosphonate (**57**) with o-aminophenol.

The reaction of **57** with o-aminophenol in oxygen flow at 160 °C results in diethyl (2-benzoxazolyl)phosphonate (**106**) [100] (Scheme 26).

**Scheme 27:**
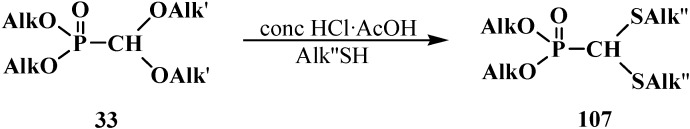
Transformation of compounds **33** into dialkyl (dialkylthiomethyl)phosphonates **107**.

When compound **33** reacted with thiols in a 1:1 mixture of acetic and hydrochloric acid at 0 °C, dialkyl (dialkylthiomethyl)phosphonates **107** were obtained in 61%–64% yield [21] (Scheme 27).

**Scheme 28:**
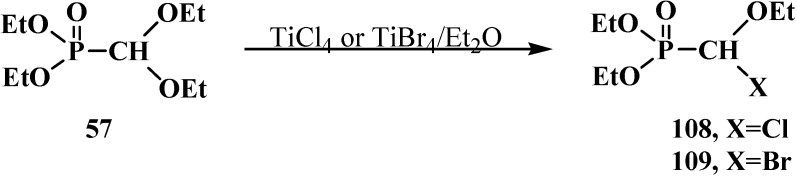
Syntheses of phosphorylated halogenacetals **108**, **109** from diethyl (diethoxymethyl)phosphonate (**57**).

Diethyl (diethoxymethyl)phosphonate (**57**) reacts with titanium tetrachloride TiCl_4_ or tetrabromide TiBr_4_ in diethyl ether to give diethyl [(ethoxy)chloromethyl]phosphonate (**108**) or diethyl [(ethoxy)bromomethyl]phosphonate (**109**) [61] (Scheme 28).

**Scheme 29:**
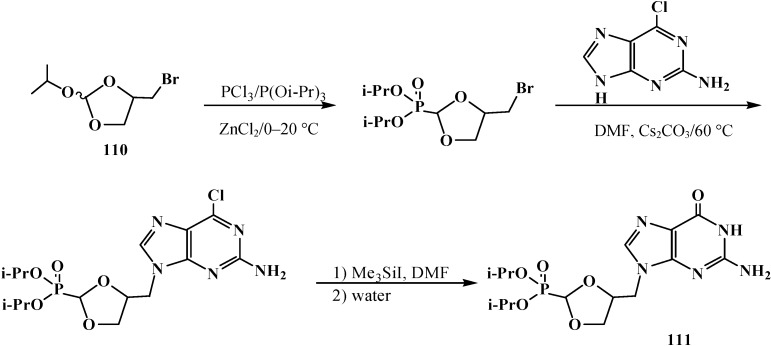
Synthesis of [1,3]-dioxolane analog guanine nucleotide **111**.

Cyclic diisopropyl [(4-bromomethyl-[1,3]-dioxolan)-2-yl]phosphonates **110** were used in the synthesis of analogs of natural purine and pyrimidine nucleotides [101]. Guanine analog **111** was prepared by the scheme (Scheme 29).

The corresponding uracil analog was obtained in a similar manner. Both compounds showed moderate activity *in vitro* toward human cytomegalovirus [101]. See also Scheme 16, Scheme 18, Scheme 19, Scheme 54, Scheme 57, Scheme 58, Scheme 60, Scheme 62, Scheme 86, Scheme 87, Scheme 88, Scheme 90, Scheme 91 and Scheme 92.

### 2.3. Phosphorylated Formaldehyde Thioacetals **14**

**Figure 5:**
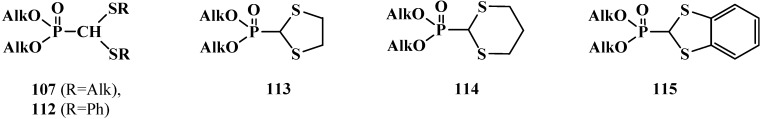
First representatives of the phosphorylated formaldehyde thioacetals **107**, **112**–**115**.

The first syntheses of compounds **14**, linear dialkyl (dialkylthiomethyl)phosphonates **107** or dialkyl (diphenylthiomethyl)phosphonates **112** [21,22], cyclic dialkyl ([1,3]-dithiolan-2-yl)phosphonates **113**, dialkyl ([1,3]-dithian-2-yl)phosphonates **114** [102] and dialkyl (1,3-benzodithiolylmethyl)phosphonates **115** [103] (Figure 5) were reported almost simultaneously in 1976–1977.

#### 2.3.1. Methods of Synthesis of Phosphorylated Formaldehyde Thioacetals **14**

**Scheme 30:**
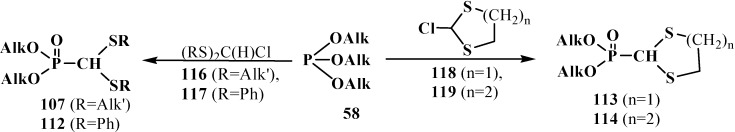
The first syntheses of linear **107**, **112** and cyclic **113**, **114** phosphorylated formaldehyde thioacetals.

The first syntheses of linear and cyclic dialkyl (dialkylthiomethyl)phosphonates **107**, **112**–**114** were performed by the analogy with the syntheses of dialkyl (dialkoxymethyl)phosphonates **33** (Scheme 9 and Scheme 13), namely, by the Arbuzov reaction of trialkyl phosphites **58** with linear (dialkylthio)chloromethanes **116** or (diphenylthio)chloromethane (**117**) [21,22], and cyclic 2-chloro-1,3-dithiolane [22,102] (**118**) or 2-chloro-1,3-dithiane (**119**) with 93%–95% yields (or quaternary ammonium salts of dimethylformamide thioacetal (only for **113**), 50%–85%) [21,22] (Scheme 30).

**Scheme 31:**
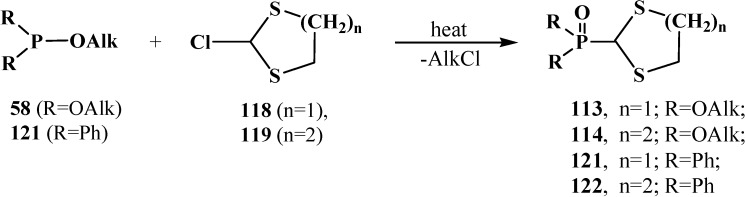
Syntheses of cyclic dialkyl (dialkylthiomethyl)phosphonates **113**, **114** and diphenyl(dialkylthiomethyl)phosphine oxides **121**, **122**.

At present, the method is used for the synthesis of cyclic dialkyl (dialkylthiomethyl)phosphonates **113**, **114**, because initial 2-chloro-1,3-dithiolane (**118**) and 2-chloro-1,3-dithiane (**119**) are readily prepared by the reaction of 1,3-dithiolane and 1,3-dithiane with *N*-chlorosuccinimide [102,104,105,106]. The method allows one to obtain in good yields both cyclic dialkyl (dialkylthiomethyl)phosphonates **113**, **114** (55%–96%), [102,104,106]) and diphenyl(dialkylthiomethyl)phosphine oxides **121**, **122** (57%–85%), [56,104,105]) (Scheme 31).

However, the poor availability of linear dialkylthiochloromethanes [21] confined the use of this method of synthesis of phosphorylated thioacetals **14** and stimulated the search for alternative methods for their synthesis.

**Scheme 32:**
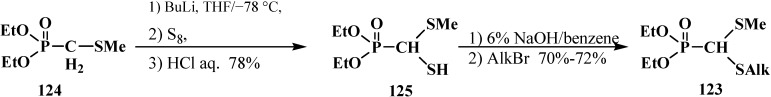
The synthesis of unsymmetrical diethyl (dialkylthiomethyl)phosphonates (**123**).

In 1979, a new two-step method was proposed for the synthesis of unsymmetrical diethyl (dialkylthiomethyl)phosphonates **123** starting from diethyl (methylthiomethyl)phosphonate (**124**). After one-pot treatment with butyllithium and elemental sulfur (sulfenylation) followed by aqueous treatment, **124** was converted into diethyl [(methylthio)mercaptomethyl]phosphonate (**125**), which under phase transfer catalysis conditions was further alkylated to give final unsymmetrical thioacetals **123** [107] (Scheme 32). In further work, alkyl halides (AlkHal) were introduced in the reaction medium immediately after sulfenylation, which enabled the preparation of thioacetals **123** by a one-pot method [108].

**Scheme 33:**
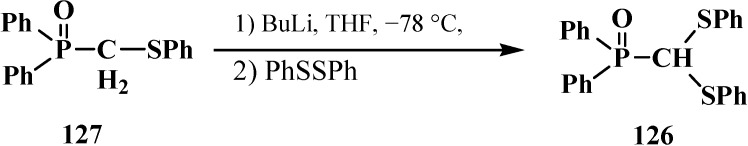
Syntheses of diphenyl(diphenylthiomethyl)phosphine oxide (**126**) from diphenyl(phenylthiomethyl)phosphine oxide (**127**).

Diphenyl(diphenylthiomethyl)phosphine oxide (**126**) [109] was previously obtained in similar manner in 75% yield from diphenyl(phenylthiomethyl)phosphine oxide (**127**) by its interaction with diphenyldisulfide (Scheme 33).

**Scheme 34:**
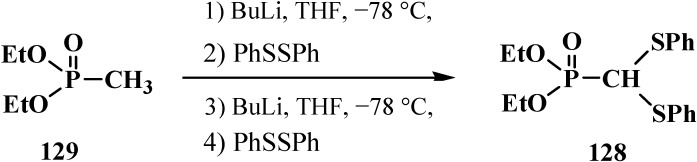
Syntheses of diethyl (diphenylthiomethyl)phosphonate (**128**) from dialkyl methylphosphonate (**129**) by a one-pot technique.

It was shown later that diethyl (diphenylthiomethyl)phosphonate (**128**) can be prepared from dialkyl methylphosphonate (**129**) [108] by a one-pot technique in 84% yield (Scheme 34).

**Scheme 35:**
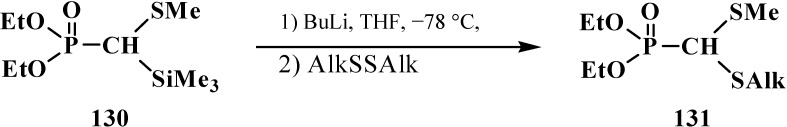
Interaction of diethyl [(methylthio)(trimethylsilyl)methyl]phosphonate (**130**) with diethyldisulfide.

Diethyl [(methylthio)(trimethylsilyl)methyl]phosphonate (**130**) undergoes a similar transformation with diethyldisulfide to form diethyl (diphenylthiomethyl)phosphonate (**131**) (yield 62%–75%) [110] (Scheme 35).

This method [107] and its variations [108,109,110] provide a possibility to synthesize linear dialkyl (dialkylthiomethyl)phosphonates **107** containing different substituents in the thioacetal group.

**Scheme 36:**
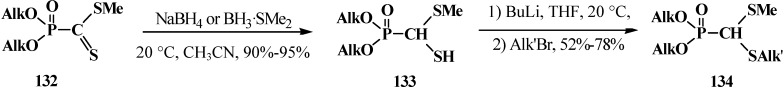
Transformation of phosphonodithioformates **132** into dialkyl [(methylthio)(alkylthio)methyl]phosphonates **134**.

The reduction of phosphonodithioformates **132** with sodium borohydride (NaBH_4_) or borane–dimethyl sulfide adduct BH_3_·SMe_2_ [111] followed by alkylation of the resulted dialkyl [(methylthio)mercaptomethyl]phosphonates **133** finally results in unsymmetrical dialkyl [(methylthio)(alkylthio)methyl]phosphonates **134** (Scheme 36).

**Scheme 37:**
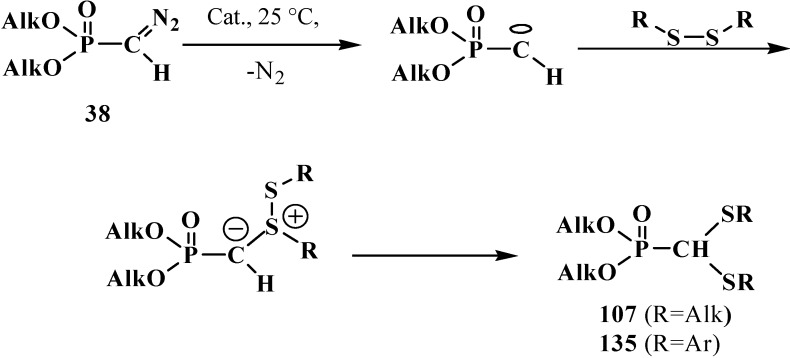
Diazomethanephosphonates **38** reaction with organic disulfides with formation of dialkyl (dialkylthiomethyl)phosphonates **107** or dialkyl (diarylthiomethyl)-phosphonates **135**.

It was shown later that dialkyl and diaryl disulfides in the presence of catalysts (Cat.) like BF_3_·Et_2_O, rhodium(II) tetraacetate Rh_2_(OAc)_4_, or copper(II) sulfate CuSO_4_, can react with diazomethanephosphonates **38**, where R=OAlk. Phosphorylated carbenes produced in the reaction undergo insertion into S–S bond to afford dialkyl (dialkylthiomethyl)phosphonates **107** or dialkyl (diarylthiomethyl)phosphonates **135** in 42%–93% yields [56,112]. Reactions proceed by the carbene mechanism (Scheme 37) [112].

**Scheme 38:**
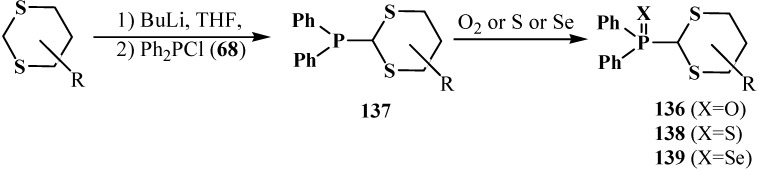
Obtaining of substituted diphenyl([1,3]-dithian-2-yl)phosphine oxides **136** and corresponding phosphine sulfides **138** and phosphine selenides **139** from dithianes.

A method of synthesis of substituted diphenyl([1,3]-dithian-2-yl)phosphine oxides **136** (yields 34%–85%) starting from substituted 1,3-dithianes and chlorodiphenylphosphine (**68**) was also suggested [104,113,114]. Initially formed diphenyl([1,3]-dithian-2-yl)phosphines **137** undergo further oxidation with molecular oxygen to final **136** (Scheme 38). Diphenyl([1,3]-dithian-2-yl)phosphine sulfides **138** or selenides **139** can be obtained by this method when elemental sulfur or selenium are used as oxidants for **137** [104,113].

The attempted preparation of **107** and **114** in one step from dialkylthiomethanes or 1,3-dithianes and dialkyl chlorophosphate in the presence of strong bases failed, as evidenced by the negligible yield of final products [21,115]. The syntheses of **107** by the interaction of thioles with dialkyl [(*N,N*-dimethylamino)alkoxymethyl]phosphonates **140** or dialkyl [(alkylthio)chloromethyl]phosphonates **141** also failed [21].

**Scheme 39:**
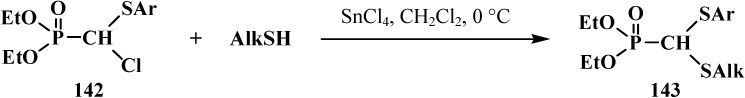
Obtaining of dialkyl [(alkylthio)(arylthio)methyl]phosphonates **143** by the reaction of dialkyl [(arylthio)chloromethyl]phosphonates **142** with thiols.

Hovewer a method of synthesis of linear phosphorylated formaldehyde thioacetals **14** by the reaction of dialkyl [(arylthio)chloromethyl]phosphonates **142** with thiols at 0 °C in the presence of equimolar amount of tin(IV) tetrachloride SnCl_4_ was succesful. This route provided a preparation of unsymmetrical dialkyl [(alkylthio)(arylthio)methyl]phosphonates **143** in 73%–84% yields [43] (Scheme 39).

Dialkyl (dialkylthiomethyl)phosphonates **107** were also obtained by the reaction of dialkyl (dialkoxymethyl)phosphonates **33** with thiols in the presence of acids [21] (see Scheme 27).

**Scheme 40:**
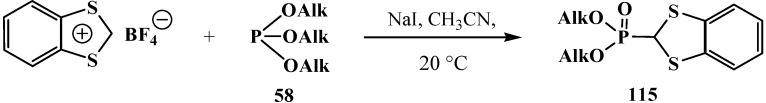
The synthesis of dialkyl (1,3-benzodithiolylmethyl)phosphonates **115** from 1,3-benzodithiolyl tetrafluoroborate.

Dialkyl (1,3-benzodithiolylmethyl)phosphonates **115** were prepared for the first time by the reaction of 1,3-benzodithiolyl tetrafluoroborate with **58** in the presence of NaI [103]. The method is used at present without changes [116] (Scheme 40).

#### 2.3.2. Chemical Properties of Phosphorylated Formaldehyde Thioacetals **14**

**Scheme 41:**
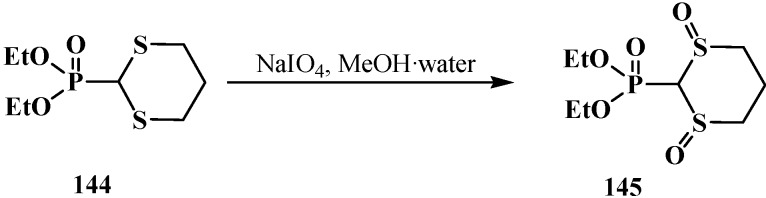
Diethyl ([1,3]-dithian-2-yl)phosphonate (**144**) oxidation by periodate.

The chemical properties of phosphorylated formaldehyde thioacetals **14** have been studied in much less detail compared with the corresponding acetals **13**. Their properties are determined by the presence of sulfur atoms and a disubstituted phosphoryl group. Both sulfur atoms are oxidized when diethyl ([1,3]-dithian-2-yl)phosphonate (**144**) is treated with sodium periodate NaIO_4_ in aqueous methanol at 20 °C [106] to yield the dioxo form **145** (Scheme 41).

**Scheme 42:**
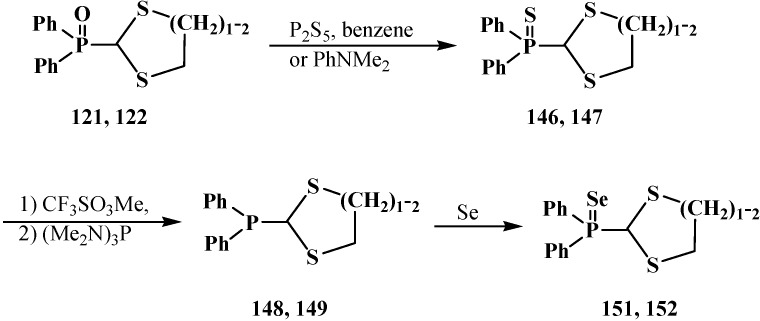
Transformation of phosphorylated cyclic thioacetals **122**, **123** into corresponding phosphines **148**, **149**, phosphine sulfides **146**, **147** and phosphine selenides **151**, **152**.

The heating of diphenyl([1,3]-dithiolan-2-yl)phosphine oxides **121** [115] and diphenyl([1,3]-dithian-2-yl)phosphine oxides **122** [56,104] with phosphorus pentasulfide P_2_S_5_ in benzene or *N,N*-diethylaniline PhNMe_2_ leads to the replacement of phosphinoyl oxygen by sulfur to yield diphenyl([1,3]-dithiolan-2-yl)phosphine sulfide (**146**) and diphenyl([1,3]-dithian-2-yl)phosphine sulfide, respectively (**147**) (Scheme 42). See also Scheme 38.

The sequential treatment of the prepared phosphine sulfides **146** and **147** with trifluoromethylsulfonate CF_3_SO_3_Me and tris(dimethylamino)phosphite (Me_2_N)_3_P finally affords diphenyl([1,3]-dithiolan-2-yl)phosphine (**148**) [105] and diphenyl([1,3]-dithian-2-yl)phosphines **149** [56,104]. The reaction of diphenyl(5-*tert*-butyl-[1,3]-dithian-2-yl)phosphine sulfide (**150**) with trichlorosilane leads to the same result [56]. The obtained phosphines **146**, **147** combine with elemental selenium to yield diphenyl([1,3]-dithiolan-2-yl)phosphine selenide (**151**) [105] and diphenyl([1,3]-dithian-2-yl)phosphine selenide (**152**) [56,104]. See also Scheme 59, Scheme 60, Scheme 64, Scheme 65, Scheme 74, Scheme 75, Scheme 76, Scheme 77, Scheme 78, Scheme 79, Scheme 80 and Scheme 81.

### 2.4. (N,N-dialkylamino)cyanomethyl Derivatives of Phosphorylated Formaldehyde (α-dialkylamino-nitriles) **15**

For 1982 till now, only two derivatives of this type of organophosphorus compounds of phosphonate series were obtained, diethyl [(*N,N*-dimethylamino)cyanomethyl]phosphonate (**153**) [24] and diethyl [(*N*-morpholino)cyanomethyl]phosphonate (**154**) [26].

#### 2.4.1. Methods of Synthesis of Diethyl [(*N,N*-Dialkylamino)cyanomethyl]phosphonates **15**

**Scheme 43:**
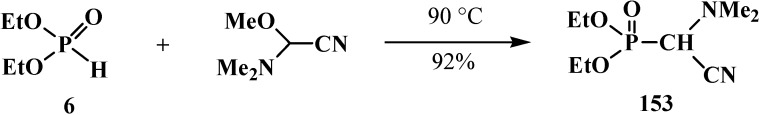
Syntheses of diethyl [(*N,N*-dimethylamino)cyanomethyl]phosphonate (**153**).

Diethyl [(*N,N*-dimethylamino)cyanomethyl]phosphonate (**153**) was obtained for the first time by a reaction similar to the preparation of phosphorylated acetals **13** (Scheme 7) from diethyl phosphite (**6**) and (*N,N*-dimethylamino)(methoxy)cyanomethane [24,25] (Scheme 43).

**Scheme 44:**
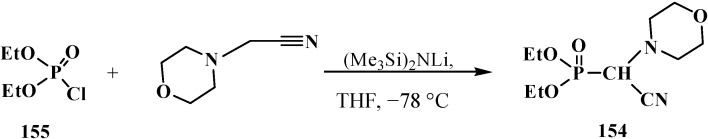
Syntheses of diethyl [(*N*-morpholino)cyanomethyl]phosphonate (**154**) from diethyl chlorophosphate (**155**).

Diethyl [(*N*-morpholino)cyanomethyl]phosphonate (**154**) was prepared by the second method from diethyl chlorophosphate (**155**) and (*N*-cyanomethyl)morpholine [26] (Scheme 44) and was used in a subsequent reaction without isolation.

#### 2.4.2. Chemical Properties of Diethyl [(*N,N*-Dialkylamino)cyanomethyl]phosphonates **15**

**Scheme 45:**
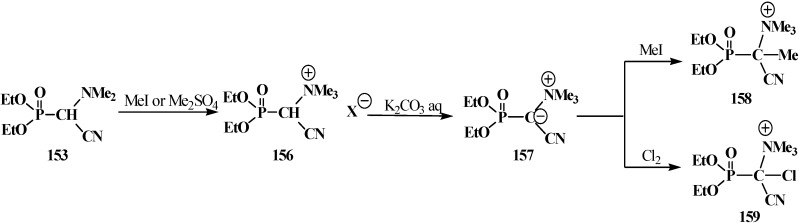
Transformation of diethyl [(*N*,*N*-dimethylamino)cyanomethyl]phosphonate (**153**).

The chemical properties of this type of compounds have been studied almost completely using the example of diethyl [(*N,N*-dimethylamino)cyanomethyl]phosphonate (**153**). The treatment of **153** with methyl iodide (MeI) or dimethyl sulfate Me_2_SO_4_ [117,118] gives rise to methylation of nitrogen atom (quaternization) of the dimethylamino group and formation of diethyl [(*N,N,N*-trimethylammonio)cyanomethyl]phosphonate cation (**156**), whose α proton shows enhanced acidity and breaks off under the action of aqueous potassium carbonate solution K_2_CO_3_. The resultant diethoxy [(*N,N,N*-trimethylammonio)cyanomethylidium]phosphonate (**157**) can react with electrophiles—MeI and molecular chlorine Cl_2_ [117], to give diethyl [(1-(*N,N,N*-trimethylammonio))(1-cyano)ethan-1-yl]phosphonate cation (**158**) and diethyl [chloro(1-*N,N,N*-trimethylammonio)cyanomethyl]phosphonate cation (**159**), respectively (Scheme 45).

**Scheme 46:**
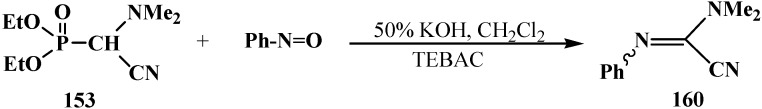
Horner—analog reaction of phosphonate **153** with nitrosobenzene.

Under phase transfer catalysis conditions—50% KOH solution, [Et_3_NCH_2_Ph]^+^Cl^−^ (TEBAC) [24] phosphonate **153** combines with nitrosobenzene PhN=O similarly to the Horner reaction to afford [(*N,N*-dimethyl)(*N'*-phenyl)amidinoyl]oxalnitrile (**160**) in 58% yield (Scheme 46). See also Scheme 55, Scheme 60, Scheme 66, Scheme 67, Scheme 82, Scheme 83, Scheme 84 and Scheme 85.

### 2.5. Diphosphinoyl N,N-Dialkylaminomethanes **16**

#### 2.5.1. Methods of Synthesis of Diphosphinoyl *N,N*-Dialkylaminomethanes **16**

**Scheme 47:**
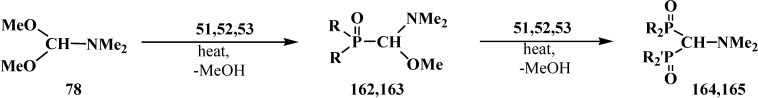
Synthesis of diphosphinoyl *N,N*-dimethylaminomethanes **164** and **165** from dimethylformamide dimethylacetal (**78**), where R, R' = OAlk, Alk, Ph. See text above.

The first report on the synthesis of tetraethyl (*N,N*-dimethylaminomethyl)diphosphonate (**161**) was published in 1968. Compound **161** was obtained in 62% yield by heating a 2:1 mixture of diethyl phosphite (**6**) and dimethylformamide dimethylacetal (**78**) [27]. Dialkyl phosphites **51**, dialkyl- and diphenylphosphine oxides **52**, **53** can be also involved in this reaction [31,32,119]. Intermediate dialkyl [(*N,N*-dimethylamino)methoxymethyl]phosphonates (**162**) or dialkyl[(*N,N*-dimethylamino)-methoxymethyl]phosphine oxides **163** may be isolated in many cases [32,119]. This allows preparation of symmetrical **164** and unsymmetrical diphosphinoyl *N,N*-dimethylaminomethanes **165** [31,32,119,120] (Scheme 47).

**Scheme 48:**
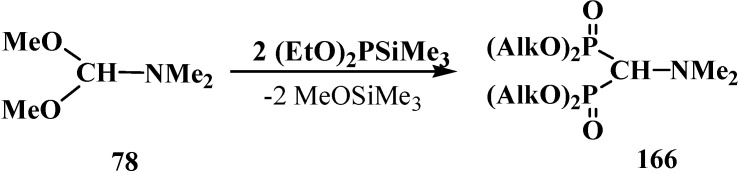
Use mixed dialkyl trimethylsilyl phosphites for the syntheses of tetraalkyl diphosphonates **166** from dimethylformamide dimethylacetal (**78**).

Other methods of synthesis of symmetrical tetraalkyl (*N,N*-dimethylaminomethyl)diphosphonates **166** were proposed later. Since trialkyl phosphites **58** do not react with dimethylformamide dimethylacetal (**78**) [18], mixed dialkyl trimethylsilyl phosphites (EtO)_2_PSiMe_3_ were successfully employed in the reaction. The reaction proceeds spontaneously at 20 °C [121] in 36%–66% yield or upon heating in the presence of zinc chloride in 72%–77% yield [122,123] (Scheme 48).

A convenient method of synthesis of unsymmetrical tetraalkyl (*N,N*-dialkylaminomethyl)- diphosphonates **167** by the reaction of trialkyl phosphites (**58**) with *N*,*N*-dialkylhalo-methylideneiminium halides [HalC(H)=NAlk_2_]^+^Hal^−^, where Hal = Cl (compounds **168**) [28,31,32,120] or Br (compounds **169**) [29] in 2:1 ratio was proposed in 1969.

**Scheme 49:**
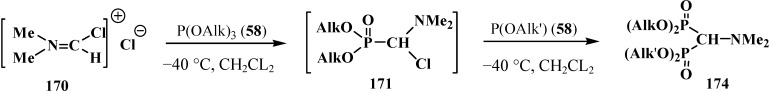
Method of synthesis of symmetrical and unsymmetrical dialkyl (*N,N*-dimethylaminochloromethyl)phosphonates **174** from *N,N*-dimethylchloromethylideneiminium chloride (**170**) and trialkyl phosphites (**58**).

It was shown that the reaction of trialkyl phosphites **58** with *N,N*-dimethylchloro- methylideneiminium chloride (**170**) proceeds via intermediate formation of dialkyl [(*N,N*-dimethylamino)chloromethyl]phosphonate **171** [31,32], specially prepared dimethyl [(*N,N*-dimethylamino)chloromethyl]phosphonate (**172**) and diethyl [(*N,N*-dimethylamino)chloromethyl]- phosphonate (**173**) may be also involved in the reaction [30,31,124]. This method enables preparation of symmetrical and unsymmetrical tetraalkyl phosphonates **174** as well (Scheme 49).

**Scheme 50:**
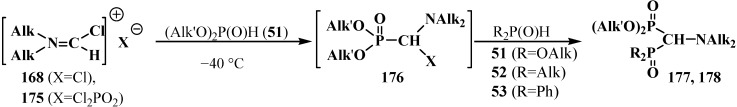
Synthesis of diphosphinoyl *N,N*-dialkylaminomethanes **177** and **178** from *N,N*-dimethylchloromethylideneiminium chloride (**170**) and hydrophosphorylic compounds **51**, **52**, **53**. See text above.

Dialkyl phosphites **51**, dialkylphosphine oxides **52** and diphenylphosphine oxide (**53**) also react with *N,N*-dialkylchloromethylideneiminium chlorides **168** [31,120] and their analogs **175** obtained from *N,N*-dialkylformamides and phosphorus oxychloride [28] (Scheme 50). The reaction also proceeds via intermediate compounds **176**, where X=Cl or Cl_2_PO_2_. This allows also the preparation of symmetrical or unsymmetrical diphosphinoyl compounds **177** and **178**, respectively.

See also Scheme 14.

#### 2.5.2. Chemical Properties of Diphosphinoyl *N,N*-Dialkylaminomethanes **16**

The chemical properties of this type of organophosphorus compounds are studied insufficiently and almost exclusively by the example of tetraethyl (*N,N*-dimethylaminomethyl)diphosphonate (**161**). Their properties are attributable to the presence of both an amino group and disubstituted phosphoryl groups.

**Scheme 51:**
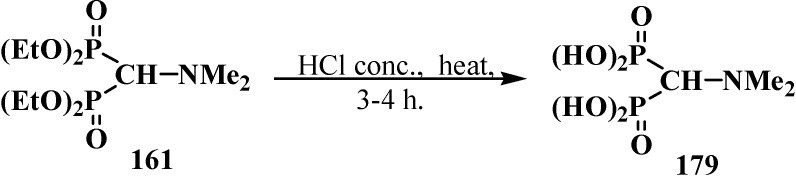
Acid hydrolysis of tetraethyl (*N,N*-dimethylaminomethyl)diphosphonate (**161**).

Hydrolysis of tetraalkyl (*N,N*-dimethylaminomethyl)diphosphonates **174** was studied using the example of compound **161**. Boiling diphosphonate **161** with concentrated hydrochloric acid leads to (*N,N*-dimethylaminomethyl)diphosphonic acid (**179**) [98,120] in almost quantitative yield (Scheme 51).

**Scheme 52:**
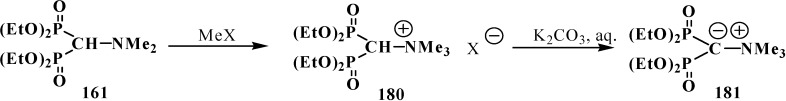
Transformation of tetraethyl (*N,N*-dimethylaminomethyl)diphosphonate (**161**) into methylidiumdiphosphonate **181**, where X = I, MeSO_3_.

The amino group of diphosphonate **161** undergoes methylation (quaternization) when reacted with methyl iodide (MeI) or dimethyl sulfate (Me_2_SO_4_) to give tetraethyl (*N,N,N*-trimethylammoniomethyl)diphosphonate cation (**180**) in 76 and 85% yields, respectively [118,125]. The α-proton of the latter shows enhanced acidity and undergoes elimination under the action of aqueous solution of potassium carbonate (K_2_CO_3_) to afford tetraethyl [(*N,N,N*-trimethylammonio)methylidium]diphosphonate (**181**) [120,125] (Scheme 52).

**Scheme 53:**
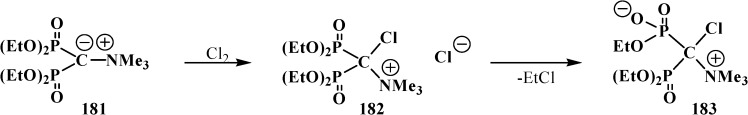
Interaction of methylidiumdiphosphonate **181** with chlorine with the subsequent formation of betaine **183**.

Ylide **181** exhibits an enhanced stability: it can be stored for a long time in air, it is thermally stable and does not react with methyl iodide [126]. However, ylide **181**, like cyanomethylide **154**, reacts with molecular chlorine to yield tetraethyl [(*N,N,N*-trimethylammonio)chloromethyl)diphosphonate chloride (**182**) that undergoes fast dealkylation on storage to afford the corresponding betaine **183** in 85% yield [120] (Scheme 53).

See also Scheme 60, Scheme 68, Scheme 72, Scheme 73, and Scheme 74.

## 3. General Chemical Properties of Phosphorylated Formaldehyde Acetals 13 and Structurally Related Compounds 14–16

### 3.1. Phosphorus–Carbon Bond Cleavage

#### 3.1.1. Cleavage of Phosphorus–Carbon Bond under the Action of Acids and Acidic Reagents

The general property of phosphorylated formaldehyde acetals **13** and structurally related compounds **14**–**16** is the possibility of phosphorus–carbon bond cleavage under the action of acids and acidic reagents [24,77,95,98], organic reagents [19,119], and bases [37,127,128].

**Scheme 54:**
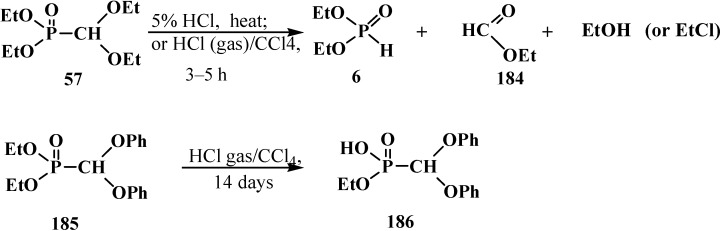
Interaction of acetals **57** and **185** with hydrochloric acid (only for **57**) and hydrogen chloride.

The cleavage of phosphorus–carbon bond under acidic conditions compounds **33** is studied on examples of diethyl (diethoxymethyl)phosphonate (**57**). Phosphonate **57** was shown to undergo cleavage of the phosphorus–carbon bond on heating with 5% hydrochloric acid [77,98] to form diethyl phosphites (**6**), ethyl formate (**184**) and ethanol. Similar degradation of compound **57** occurs in a flow of hydrogen chloride at 20 °C [95] (ethyl chloride is formed as a byproduct) (Scheme 54). Unlike **57**, interaction of diethyl (diphenoxymethyl)phosphonate (**185**) with dry hydrogen chloride does not lead to the cleavage of phosphorus–carbon bond, but leads to the dealkylation of one ethoxy substituent at the phosphorus atom with obtaining ethyl (diphenoxymethyl)phosphonic acid (**186**) (Scheme 54).

**Scheme 55:**
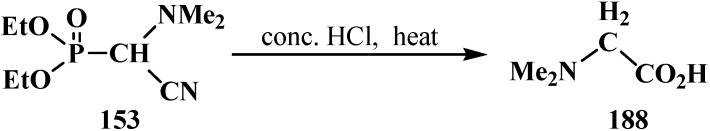
Obtaining *N,N*-dimethylaminoacetic acid (**188**) by acid hydrolysis of cyanomethylphosphonate **153**.

It was shown that acid hydrolysis of diethyl (diethylthiomethyl)phosphonates (**187**) is accompanied by a partial cleavage of the phosphorus–carbon bond [98]. Prolonged refluxing of diethyl [(*N,N*-dimethylamino)cyanomethyl]phosphonate (**153**) in concentrated hydrochloric acid leads to *N,N*-dimethylaminoacetic acid (**188**) in 87% yield [24] (Scheme 55).

**Scheme 56:**
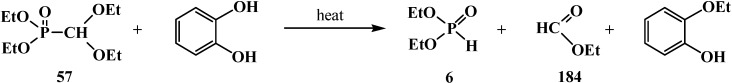
Destruction of acetales **57** at its interaction with catechol.

It was shown in [95] that the attempted transesterification of ethoxy substituents at the phosphorus atom of phosphonate **57** by catechol residue also leads to the cleavage of the phosphorus–carbon bond (Scheme 56).

The cleavage of phosphorus–carbon bond may also occur when diethyl (dialkoxymethyl)phosphonates **93** are exposed to phosphorus pentachloride [95].

#### 3.1.2. The Cleavage of Phosphorus–Carbon Bond in Reactions with Organic Coreactants

**Scheme 57:**
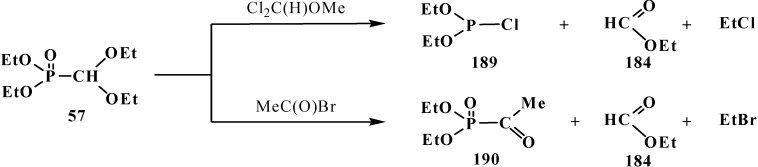
Acetales **57** interaction with Cl_2_C(H)OMe and MeC(O)Br leading to the cleavage of phosphorus–carbon bond.

The reactions of **57** with dichloromethoxymethane Cl_2_C(H)OMe or acetyl bromide MeC(O)Br was shown to be accompanied by the cleavage of phosphorus–carbon bond [19] to form diethoxychlorophosphine (**189**) and diethyl acethylphosphonate (**190**) (Scheme 57).

The reaction of diethyl phosphite (**6**) with dipropyl [(*N,N*-dimethylamino)ethoxymethyl]phosphonate (**191**) is also accompanied by the partial cleavage of the phosphorus–carbon bond [119].

#### 3.1.3. Phosphorus–Carbon Bond Cleavage under the Action of Bases

**Scheme 58:**
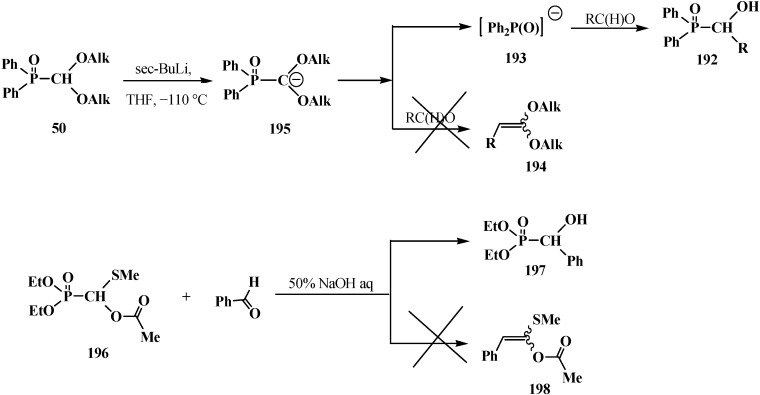
Cleavage of phosphorus–carbon bond of acetal **57** and O,S-acetal **196** leading to the formation of α-phosphorylated alcohols **192** and **198**.

The reaction of the lithium derivatives of diphenyl(dialkoxymethyl)phosphine oxides **50** with *n*-octanal and *p*-isopropylbenzaldehyde leads to α-phosphorylated alcohols **192**, the products of addition of diphenylphosphinite anion (**193**) to the carbonyl group (Abramov reaction) [127,128], rather than ketene *O,O*-acetals **194** as expected products of the Horner reaction [9,10] (Scheme 58). The reason of this course is the instability of diphenyl (dialkoxymethyl)phosphine oxide anion (**195**) that compound **50** decomposes after long exposition under the reaction conditions to form anion **193**, which further undergoes addition to the aldehyde carbonyl group [127,128].

Like phosphine oxides **50**, the reaction of diethyl [(acetoxy)methylthiomethyl]phosphonate (**196**) with benzaldehyde PhC(H)O under phase transfer catalysis conditions gives rise to diethyl [(hydroxy)phenylmethyl]phosphonate (**197**), the Abramov reaction product, instead of the expected ketene O,S-acetal **198** resulting from the Horner reaction [37] (Scheme 58).

**Scheme 59:**
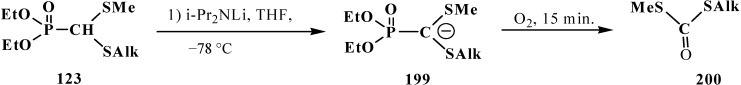
Destruction of thioacetal **123** in the presence of oxygen, leading to formation of dithiocarbonates **200**.

Lithiated anions **199** of diethyl [(methylthio)(alkylthio)methyl]phosphonates **123** react quickly with molecular oxygen with the cleavage of phosphorus–carbon bond and formation of dialkyl dithiocarbonates **200** (yields 71%–72%) [129] (Scheme 59).

See also Scheme 63, Scheme 96, Scheme 103, Scheme 104, Scheme 113, Scheme 114, Scheme 115, Scheme 116, Scheme 117 and Scheme 120.

### 3.2. Synthesis of Formacetalphosphonic Acids

Attempted preparations of formacetalphosphonic acids **201**, **202** and **203** from the corresponding phosphorylated acetal **57**, thioacetal **187** and aminonitrile **153** by acid hydrolysis lead to cleavage of the phosphorus–carbon bond, but in the case of compound **91** with a aromatic substituent in the acetal group, it undergoes acid hydrolysis to give the expected phosphonic acid **92** (Scheme 20). See also Section 3.1.1. “Cleavage of phosphorus–carbon bond under the action of acids and acidic reagents”.

**Scheme 60:**
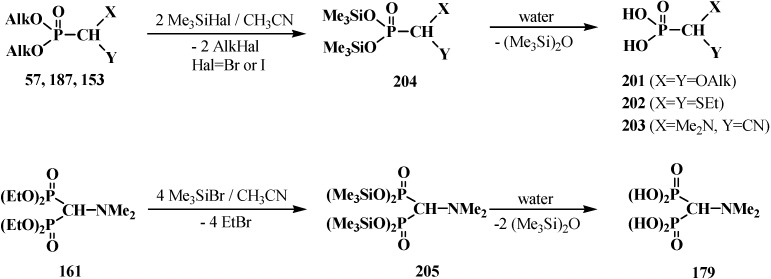
Syntheses of formacetalphosphonic acids **201**–**203** and **179** from compounds **57**, **187**, **153**, **161** by means of Me_3_SiBr or Me_3_SiI. See text above.

However, the target formacetalphosphonic acids **201**–**203** may be successfully prepared by the reaction of acetals **33**, diethyl (diethylthiomethyl)phosphonate (**187**), diethyl [(*N,N*-dimethyl-amino)cyanomethyl]phosphonate (**153**), and tetraethyl(*N,N*-dimethylaminomethyl)diphosphonate (**161**) with trimethylsilyl bromide (Me_3_SiBr) in acetonitrile. Alkyl groups at the phosphorus atom are eliminated as alkyl halides to afford the corresponding intermediate bis(trimethylsilyl) phosphonates **204** (or tetrakis(trimethylsilyl) phosphonate (**205**) from diphosphonate **161**), which are further readily hydrolyzed by water treatment to give:

‒ (dialkoxymethyl)phosphonic acids **201** in 93%–100% yield from **33** [19,98]. It was shown that the reaction **33** with an equimolar mixture of trimethylsilyl chloride Me_3_SiCl and NaBr or LiBr [130] in acetonitrile of trimethylsilyl chloride and NaI in methylene chloride [131] leads to the same result,
‒ (diethylthiomethyl)phosphonic acid (**202**) from **187** in 58% yield [98],
‒ (1-dimethylamino-1-cyanomethyl)phosphonic acid (**203**) from **153** in 55% yield [24],
‒ (*N,N*-dimethylaminomethyl)diphosphonic acid (**179**) from **161** in 65% yield [98] (Scheme 60).

See also Scheme 15, Scheme 20, Scheme 21, Scheme 22, Scheme 29 and Scheme 54.

### 3.3. Alkylation (Acylation) of the Formacetal Carbon Atom

**Scheme 61:**
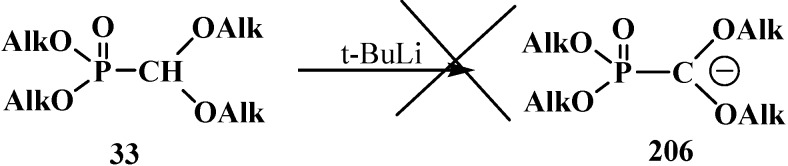
Phosphorylated acetals **33** do not produce carbanions **206**.

Dialkyl (dialkoxymethyl)phosphonates **33** produce no stable phosphorylated carbanion **206** when reacted with bases (no metallation occurs, even under the action of *tert*-butyllithium (*t*-BuLi), which provides no possibility for further alkylation and acylation of the formacetal group [23,132] (Scheme 61).

**Scheme 62:**
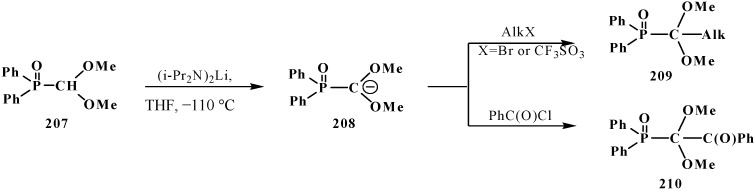
Alkylation and acylation of carbanion **207**.

This fact was explained by insufficient stabilization of the negative charge of carbanion on the two oxygen atoms in the α-position [23]. However, it was shown in 1983 [133] that, in contrast to phosphonates **33**, diphenyl(dialkoxymethyl)phosphine oxides **50** produce phosphorylated anions **195** at −110 °C that undergo metallation. The reason for the stability of the lithium derivatives of phosphine oxides **50** is the ability of diphenylphosphinoyl group to delocalize the negative charge of carbanion **195** [20,134] (Scheme 58). By the example of anion **207** of diphenyl(dimethoxy-methyl)phosphine oxide (**208**), it was shown that it is rather stable to subsequent alkylation with alkyl halides and acylation with benzoyl chloride [20]. The reactions afford diphenyl[(dimethoxy)alkylmethyl]phosphine oxides **209**, in 30%–94% yields, and diphenyl-[(dimethoxy)benzoylmethyl]phosphine oxide (**210**) (60%) (Scheme 62).

**Scheme 63:**
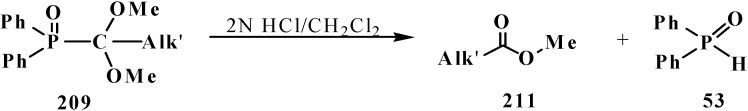
Hydrolysis of phosphine oxide **209** leads to phosphorus–carbon bond cleavage.

In acidic medium at 20 °C, phosphine oxides **209** are readily decomposed with cleavage of the phosphorus–carbon bond. The resultant methyl carboxylates **211** are homologous to the initial alkyl halides—carbon chain elongation by one atom (Scheme 63).

Similarly, according to ^1^H-NMR spectroscopy, the methanolysis of diphenyl[(1,1-dialkoxy)nonan-1-yl)phosphine oxide (**212**) in the presence of trifluoroacetic acid leads to 1,1,1-trimethoxynonane (**213**) in 65% yield [20].

Nonetheless, the storage of a solution of lithiated anion **207** for two hours even at −110 °C causes the cleavage of phosphorus–carbon bond (see Scheme 58).

**Scheme 64:**
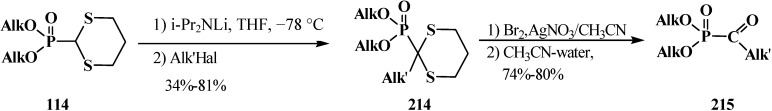
Thioacetals **114** alkylation with the subsequent transformation of thioketals **214** into α-phosphorylated carbonyl compounds **215**.

Distinct from acetals **33** [23,132], the two sulfur atoms of thioacetals **113**, **114** stabilize well the neighboring carbanion [23], therefore the α-hydrogen atom in the thioacetal group is readily removed under the action of strong bases [23,132] in both dialkyl (dialkythiomethyl)phosphonates **113**, **114** [135] and diphenyl(dialkythiomethyl)phosphine oxides **121**, **122** [113]. Further, the carbanions are readily alkylated with alkyl halides [113,135]. For example, dialkyl ([1,3]-dithian-2-yl)phosphonates **114** in this reaction produce dialkyl [(2-alkyl-[1,3]-dithian)-2-yl]phosphonates **214**, and their oxidative decomposition may result in α-phosphorylated carbonyl compounds **215** (Scheme 64).

**Scheme 65:**
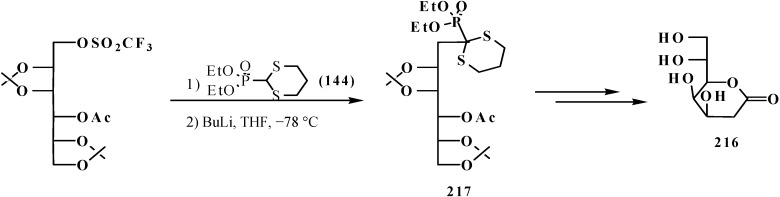
Synthesis of 3-deoxy-d-manno-octulosonic acid (**216**) by the alkylation of thioacetal **144**.

The possibility to alkylate diethyl ([1,3]-dithian-2-yl)phosphonate (1**44**) was used for the elongation of the hydrocarbon chain in the synthesis of 3-deoxy-D-manno-octulosonic acid (**216**), through compound **217** as alkylated form **144** [136] (Scheme 65).

**Scheme 66:**
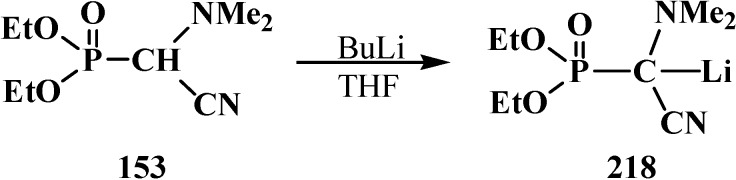
Synthesis of stable lithium derivative **218** of diethyl [(*N,N*-dimethylamino)- cyanomethyl]phosphonate (**153**).

In contrast to dialkyl (dialkoxymethyl)phosphonates **33** and similarly to phosphorylated formaldehyde thioacetals **14**, diethyl [(*N,N*-dimethylamino)cyanomethyl]phosphonate (**153**) is readily deprotonated under the action of sodium hydride in dioxane or dimethyl sulfoxide or 50% KOH solution under phase transfer catalysis conditions [24] as well as with butyllithium BuLi in THF [25]. Lithium derivative **218** of diethyl [(*N,N*-dimethylamino)cyanomethyl]phosphonate (**153**) proved to be so stable that it could be stored without decomposition for several months [25] (Scheme 66).

**Scheme 67:**
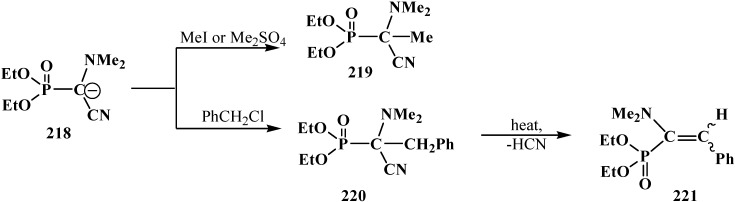
Anion **218** alkylation.

The anion of **218** undergoes alkylation at the carbon atom when treated with methyl iodide MeI [24,25,117], dimethyl sulfate Me_2_SO_4_ [117] or benzyl chloride PhCH_2_Cl [24] to give α-alkylated derivatives **219** and **220**. Benzyl derivative **220** prepared by this method eliminates hydrogen cyanide on heating to give α-phosphorylated enamine **221** [24] (Scheme 67).

**Scheme 68:**
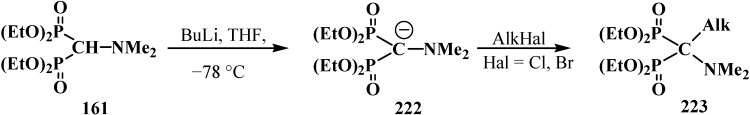
Alkylation of methyldiphosphonate **161**, (Hal = Cl, Br).

Similarly to diphenyl(dialkoxymethyl)phosphine oxides (**50**), cyclic dialkyl (dialkylthiomethyl)- phosphonates **113**, **114**, cyclic diphenyl(dialkylthiomethyl)phosphine oxides **121**, **122**, and diethyl [(*N,N*-dimethylamino)cyanomethyl]phosphonate (**153**), and tetraethyl (*N,N*-dimethylaminomethyl)- diphosphonate (**161**) under the action of strong bases readily eliminate a proton from the acetal carbon atom to give anion **222**. This provides an opportunity for its further alkylation (compounds **223**) [28], which has been used in the synthesis of pesticides (Scheme 68).

### 3.4. Horner Reaction

**Scheme 69:**
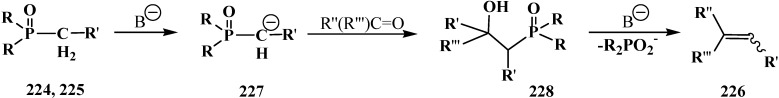
Syntheses of olefins **226** from aldehydes and ketones by means of Horner’s reaction, R = OAlk, Ph; R' = Alk; R'', R''' = H, Alk, Ar.

In 1958 and 1959 L. Horner and co-authors reported their discovery of a new reaction [137,138] that they named as “P=O-activated olefination”. The authors showed that the reaction of alkyl(diphenyl)phosphine oxides **224** and dialkyl alkylphosphonates **225** with aldehydes and ketones in the presence of strong bases produce olefins **226** (Scheme 69). Key reaction intermediates—lithium derivatives of carbanion of the initial phosphinoyl compounds **227** and β-phosphorylated hydroxy derivatives **228** were identified on the example reaction of benzyl(diphenyl)phosphine oxide (**229**) with benzaldehyde in the presence of phenyllithium [138].

The reaction has a number of advantages in comparison with similar reaction of phosphorus ylides previously described by L. Wittig [139] where ketones are difficult to react, whereas both aldehydes and ketones undergo the Horner reaction. It was further shown that Horner reaction has a larger synthetic potential and is applicable for the synthesis of other types of organic compounds, for example, allenes, cyclopropanes, terminal [140] and disubstituted alkynes [132]. The involvement of phosphonates functionalized at the α-position with dialkylamino, alkoxy or alkylthio groups in the reaction leads to enamines, vinyl ethers [132,141,142,143] and vinyl thioethers [141,143]. Their subsequent hydrolysis affords aldehydes and ketones with elongated hydrocarbon chain in high yields (homologation).

**Scheme 70:**
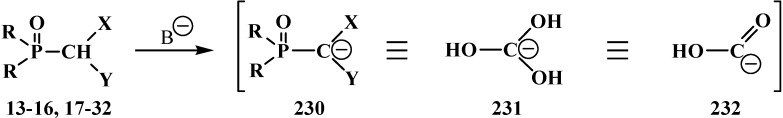
Compounds **13**–**16**, **18**–**32** as hidden form of reversed-polarity formate carbanion **232**, where R = OAlk, Ph; X, Y = AlkO, AlkS, Alk_2_N, CN, R_2_P(O), Hal, AlkS(O).

The Horner reaction also provides the possibility to prepare carboxylic acids homologized by one carbon atom via the shortest route starting from phosphinoyl compounds functionalized at the α-position with two heteroatoms, namely, phosphorylated formaldehyde acetals and structurally related compounds [132,141]. In this case, carbanions **230** of phosphorylated formaldehyde acetals and structurally related compounds **13**–**16**, **18**–**32** behave as a masked form of triply functionalized carbanions **231** that may be considered as a synthetic equivalent or carrier of reversed-polarity formate carbanion [O=C–OH]^−^ **232** [20,141] (Scheme 70).

**Scheme 71:**
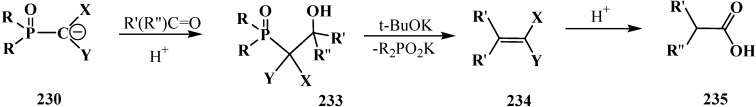
Syntheses of carboxylic acids **235** by means of Horner's reaction, R',R'' = H, Alk, Ar.

Carbanions **230**, prepared by the deprotonation of the initial phosphoryl compound, react with carbonyl compounds to afford β-phosphorylated alcohols **233**, which can be isolated. The subsequent treatment of alcohols **233** with strong bases, usually potassium *tert*-butoxide, leads to ketene acetals and structurally related compounds **234** that are valuable precursors in the synthesis of organic compounds of different kinds [23,24,132,133,143]. Further acid hydrolysis of compounds **234** produces carboxylic acids **235** (Scheme 71) or their derivatives, for example esters **236**, or thioesters **237**, depending on the conditions.

However, the simplest and most available phosphorylated formaldehyde acetals, dialkyl (dialkoxymethyl)phosphonates **33**, do not form stable carbanions [23,132], therefore the attempted synthesis of carboxylic acids and their derivatives by Horner reaction failed for a long time. Among acetals of phosphonate type compounds, only diethyl (5,6-dichloro-1,3-benzodioxomethyl)phosphonate (**91**) participated in the reaction with ketones at 90 °C in dioxane in the presence of sodium hydride NaH to give ketene acetals in 19%–32% yields [23]. See also Section 3.3 “Alkylation of formacetal carbon atom”.

**Scheme 72:**
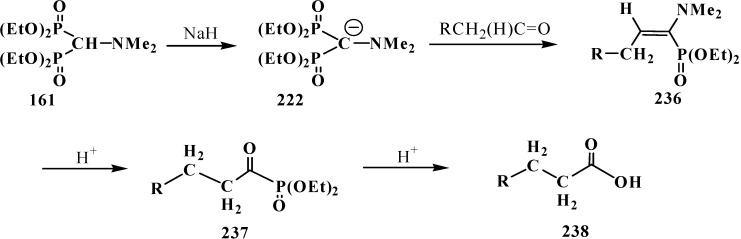
Synthesis of carboxylic acids **238** by reacting tetraethyl (*N,N*-dimethylaminomethyl)diphosphonate (**161**) with aliphatic and aromatic aldehydes.

Carboxylic acids were obtained for the first time by Horner reaction in 75%–90% yields in 1968 by reacting tetraethyl (*N,N*-dimethylaminomethyl)diphosphonate (**161**) with aliphatic and aromatic aldehydes [27]. After formation of the carbanion **222**, the reaction proceeds through the sequential formation of 1-dimethylaminoalkenylphosphonates—α-phosphorylated enamines **236**, then α-phosphinoylacyl derivatives **237**, and finally yields free linear acids **238** (Scheme 72).

**Scheme 73:**
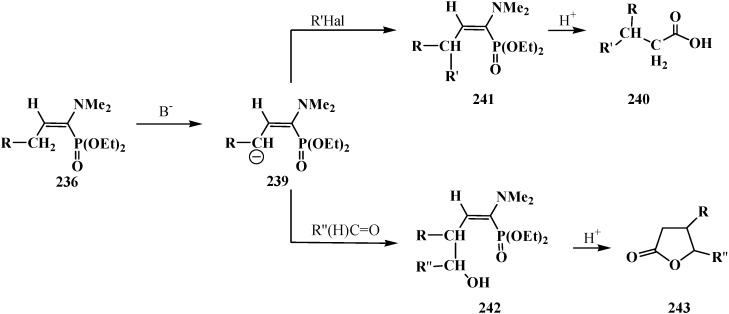
Syntheses of carboxylic acids **240** and γ-butyrolactones **243** from phosphorylated enamines **236**.

Since phosphorylated enamines **236** (synthesized from aliphatic aldehydes only) contain an anion-stabilizing diethoxyphosphinoyl group in the α-position, the methylene group in the γ-position is readily deprotonated under the action of strong bases. The resulting phosphorylated aminoallyl anions **239** react with alkyl halides and finally form carboxylic acids **240** branched at the β-position via the phosphorylated enamines **241**. The reaction of anions **239** with aldehydes gives rise to hydroxy compounds (**242**) and then to β,γ-disubstituted γ-butyrolactones **243** over unisolated γ-hydroxy carboxylic acids that undergo fast cyclization under the reaction conditions [144] (Scheme 73).

**Scheme 74:**
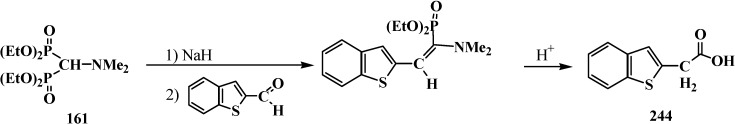
Synthesis of 2-benzothienylacetic acid **244** by Horner’s reaction.

Because only aldehydes react with compound **161** [27,132], this method of synthesis of carboxylic acids is not widely used. However, compound **161** is employed for the preparation of substituted acetic acids as intermediate stages in the synthesis of potential pharmaceuticals [124,145] and pesticides [28], for example, acid **244** (Scheme 74).

**Scheme 75:**
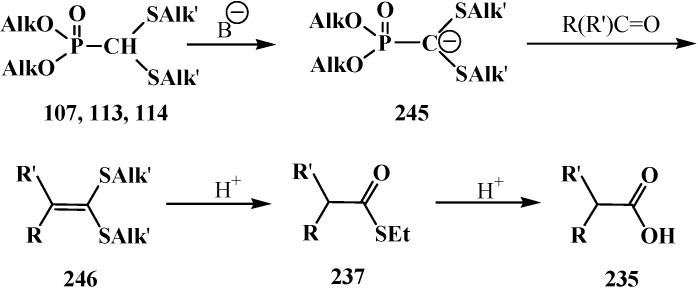
Syntheses of carboxylic acids **235** by reacting thiomethylphosphonates **107**, **113**, **114** with aldehydes and ketones.

The successful homologation of aldehydes with the use of diphosphonate **161** stimulated further search for the synthetic equivalents of formate carbanion **232** among organophosphorus compounds. In 1976–1977, linear (**107**) and cyclic dialkyl (dialkythiomethyl)phosphonates **113**, **114** were proposed [21,146]. These compounds can form a stable carbanion **245**, and react with both aldehydes and ketones to form ketene thioacetals **246** and further under subsequent hydrolysis (over thioesters **237**) produce homologous carboxylic acids **235** (Scheme 75), see also section “Alkylation of formacetal carbon atom”. Ketene thioacetals **246** were obtained from ketones and aldehydes [102,146], including those unsaturated, in 66%–82% and 80%–96% yields, respectively, as mixtures of *Z*/*E* isomers [23].

**Scheme 76:**
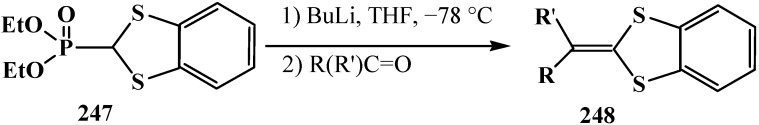
Syntheses 1,4-benzodithiafulvenes **248** from diphenyl([1,3]-dithian-2-yl)phosphine oxide (**122**) by the reaction with carbonyl compounds.

The reaction is observed also for diphenyl([1,3]-dithian-2-yl)phosphine oxide (**122**) [123,124] and diethyl (1,3-benzodithiolylmethyl)phosphonate (**247**) [113,147]. Phosphonate **247** produces benzo-analogs of ketene thioacetals, 1,4-benzodithiafulvenes **248**, in 92%–98% yields when reacted with carbonyl compounds (Scheme 76).

**Scheme 77:**
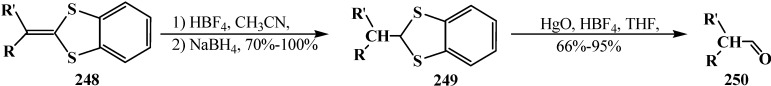
Obtaining branched aldehydes **250** from 1,4-benzodithiafulvenes **248**.

Phosphorylated thioacetals **107**, **113**, **114** and thioacetals produced from its ketene **246**, **248** have a synthetic importance because, along with carboxylic acid synthesis, they undergo numerous reactions to afford various products [23]. For example, aldehydes **250**, branched out in α-position are formed in the reduction of 1,4-benzodithiafulvenes **248** followed by hydrolysis (over the stage of reduced compounds **249**) [148] (Scheme 77).

**Scheme 78:**
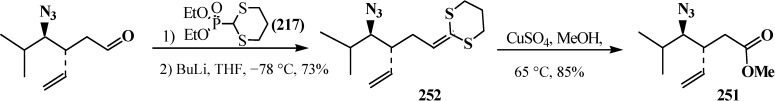
Synthesis of methyl esters **251** by methanolysis of thioacetal **252**.

Methyl esters **251** result from thioacetal **252** methanolysis [149] (Scheme 78).

**Scheme 79:**
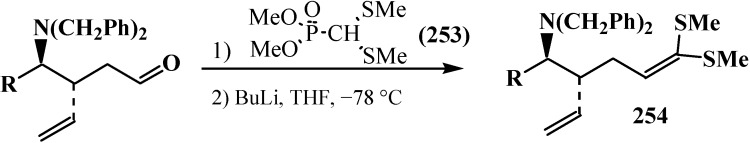
Synthesis of compound **254**—an intermediate stage of synthesis of dipeptide mimetics.

**Scheme 80:**
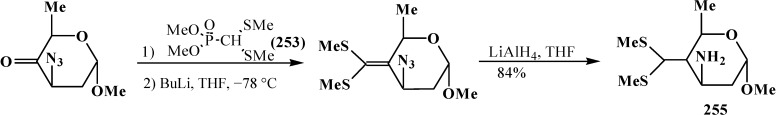
Synthesis of the antibiotic thienamycin (**255**) by means of thiomethylphosphonate **253**.

**Scheme 81:**
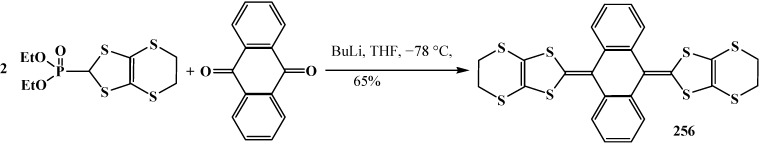
Example of synthesis of “organic metals” **256**.

Dimethyl (dimethylthiomethyl)phosphonate **253** is used in the practice of contemporary organic chemistry, for example, in the intermediate stages of synthesis of biologically active dipeptide mimetics **254** [149] (Scheme 79), the antibiotic thienamycin (**255**) [150] (Scheme 80), and “organic metals” **256** [116,151,152,153,154], for example (Scheme 81).

However, the long duration of the two-stage conversion of ketene thioacetals **246** into acids **235**, often in the presence of mercury Hg^2+^ [141] or copper Cu^2+^ salts [149] and the necessity of working with mercaptans [23,141,154] limits the application of phosphorylated formaldehyde thioacetals **14** in the Horner reaction.

Therefore, the search for efficient precursors for the synthesis of carboxylic acids **235** from carbonyl compounds by the Horner reaction has continued. From the mid-1970s to the early 1980s, many acetal-like derivatives of diethyl formylphosphonates **13**–**32** [18,19,21,22,23,24,25,26,27,28,29,30,31,32,33,34,35,36,37,38,39,40,41,42,43,44,45,46,47,48,49,50,51,52,53,54,55,56], where the negative charge of the carbanion was stabilized by two heteroatoms of the “acetal” group, were obtained [54]. See also Figure 2, where R = OEt. In the case of X = Me_3_Si, Peterson olefination prevails over the Horner reaction [54,55,56,110] and trimethylsyloxy fragment is a leaving group.

Because the majority of compounds **18**–**32** have no substantial advantages over the phosphorylated formaldehyde thioacetals **14**, the study of Horner reaction with their participation was confined mainly to academic interest. More detailed studies of reactivity of the majority of these compounds were not conducted.

Among the compounds synthesized over this period, diethyl [(*N,N*-dialkylamino)cyano- methyl]phosphonate (**153**) and diphenyl(dialkoxymethyl)phosphine oxides **50** were involved in the practice of organic synthesis.

**Scheme 82:**
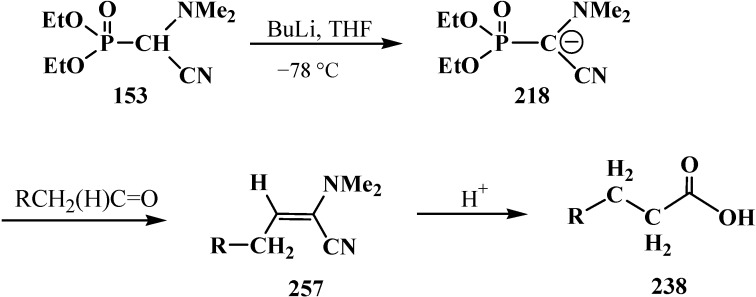
Carboxylic acids **238** synthesis by means of diethyl [(*N,N*-dialkylamino)cyanomethyl]phosphonates **153**, where R = Alk, Ph.

Compound **153** proposed in 1982 [24] reacts like dialkyl (dialkylthiomethyl)phosphonates **107** with aldehydes, in 50%–69% yields, and acetophenone as ketone example, in 24% yield [24,132]. The products of Horner reaction in this case are cyanoenamines **257**, whose acid hydrolysis produces linear carboxylic acids **238** homologous to the initial carbonyl compounds [24,25,132] (Scheme 82). See also Scheme 72.

**Scheme 83:**
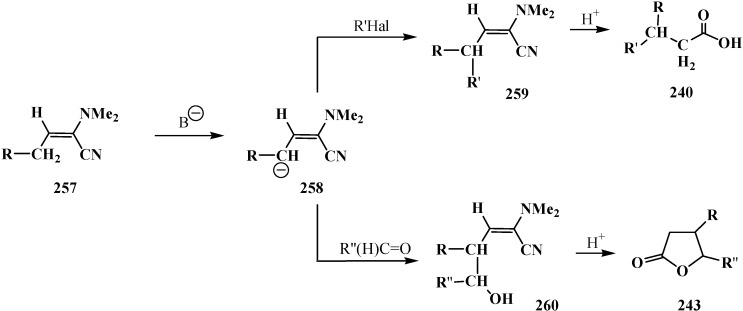
Enamines **257** alkylation by means of alkyl halides or aldehydes leads to obtaining branched acids **240** or butyrolactones **243**.

Like phosphorylated enamines **236** (Scheme 73), compound **257** contains an anion-stabilizing CN-group in the α-position. Resulting cyanoaminoallyl anions **258** combine with alkyl halides to form carboxylic acids **240** through cyanoenamines **259**, while the reaction with aldehydes leads to γ-hydroxy acids that undergo cyclization to give β,γ-disubstituted γ-butyrolactones **243** through cyano aminoallylalcohols **260** [24,25,132,144] (Scheme 83).

**Scheme 84:**
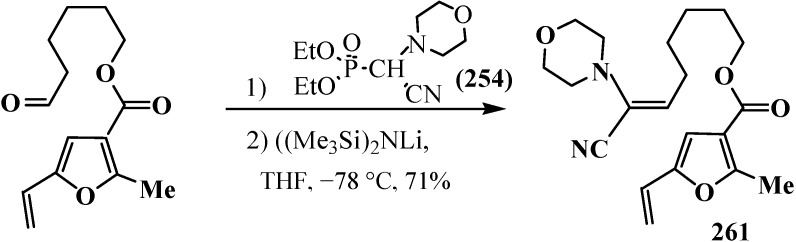
Synthesis of colerofragarone fragment **262** using diethyl [(*N*-morpholino)cyanomethyl]phosphonate (**154**).

Diethyl [(*N*-morpholino)cyanomethyl]phosphonate (**154**) was used in the synthesis of the colerofragarone fragment—terminator of fungi *Collerotrichum fragariac* [155] (Scheme 84, compound **261**).

**Scheme 85:**
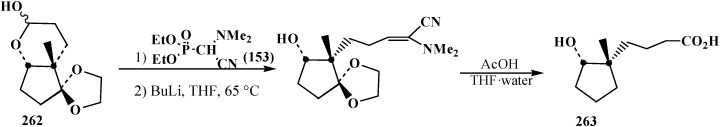
Synthesis of **263**, a semi-product of synthesis of the analog of cloprosterol PGF_2_ from cyclic semiacetal **262** by means of phosphonate **153**.

It was shown that compound **153** can react also with cyclic semiacetal **262**, which was used in one of the stages of synthesis of prostaglandin analog cloprosterol PGF_2_ [155,156] (Scheme 85, compound **263**).

**Scheme 86:**
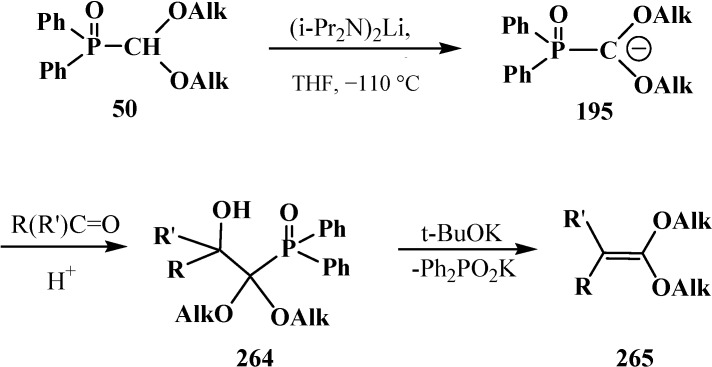
Synthesis of ketene acetals **265** from diphenyl(dialkoxymethyl)phosphine oxides **50** through conpounds **264** as intermediates.

However, compound **153** was not widely used because of the low yields in its reactions with ketones [25] and formation of hydrogen cyanide on hydrolysis of cyanoenamines. Almost simultaneously with **153**, a successful synthesis of ketene acetal at −110 °C starting from diphenyl(dialkoxymethyl)phosphine oxides **50**, over carbanion **195** and both aldehydes and ketones was reported in 1983 [133]. Intermediate β-hydroxydiphenylphosphinoyl derivatives **264** were isolated in almost quantitative yield that further react with potassium *tert*-butoxide (*t*-BuOK) to give ketene acetals **265** in 45%–85% yields [133] (Scheme 86).

**Scheme 87:**
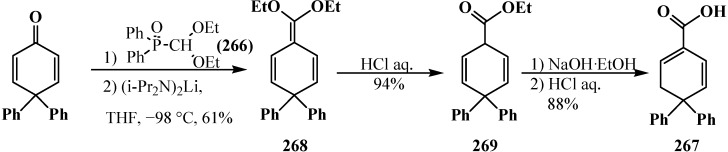
Synthesis of carboxylic acid **267** from (diethoxymethyl)phosphine oxide (**266**) through ketene acetal **268** and ethyl ester **269**.

This publication attracted no attention for a long time, although in 1993 it was shown that the reaction of diphenyl(diethoxymethyl)phosphine oxide (**266**) with substituted cyclohexadienone in the presence of lithium diisopropylamide leads to the formation of carboxylic acid **267** through the stages of ketene acetal **268** and ethyl ester **269** formation [20,157] (Scheme 87).

**Scheme 88:**
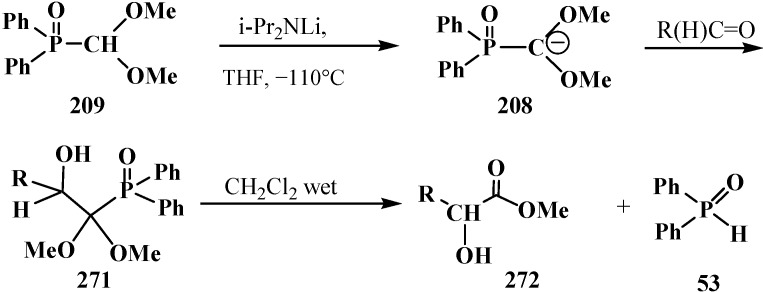
Synthesis and decomposition of β-hydroxydiphenylphosphinoyl compounds **271** lead to methyl esters of α-hydroxycarboxylic acids **272**.

It was found in 2002, however, that the reaction of diphenyl(dimethoxymethyl)phosphine oxide (**209**) with aliphatic aldehydes, in contrast to other phosphorylated equivalents of formate anion—compounds **107**, **112**–**115**, **153** (**154**), **161** (**166**), may also result in derivatives of α-hydroxycarboxylic acids **270** [140,158,159]. Initially formed β-hydroxydiphenylphosphinoyl derivatives **271** in wet acidified dichloromethane readily decompose with the cleavage of the P–C bond to afford methyl esters of α-hydroxycarboxylic acids **272** in 41%–89% yield and diphenylphosphine oxide (**53**) (Scheme 88).

**Scheme 89:**
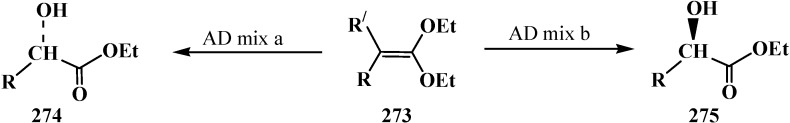
Using of Sharpless asymmetric dihydroxylation (AD reaction) for synthesis of chiral α-hydroxycarboxylic acids **274**, **275** from ketene acetals **273**.

It was further shown that ketene acetals **273** resulting from aldehydes and phosphine oxide **266** may be successfully oxidized [20,128,160] in the Sharpless asymmetric dihydroxylation—AD reaction [161]. As a result, the esters of chiral α-hydroxycarboxylic acids **274**, **275** were obtained in 49%–94% yields and enantiomeric excess up to 98% (Scheme 89).

**Scheme 90:**
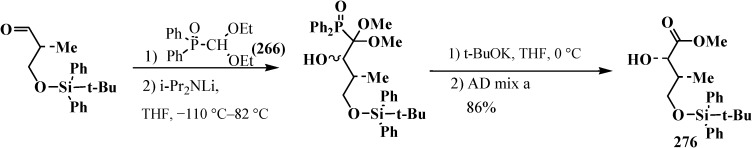
Using of combination of Horner and AD reactions to obtain compound **276**—intermediate stage of synthesis of the diterpenoid tonantzitlolone.

It should be noted that ketene thioacetals **246** do not undergo the AD reaction. Mixed *O,S*-acetal **18** reacts but yields and enantiomeric excess of α-hydroxycarboxylic acids do not exceed 7%–37% and 80%, correspondingly [127,128]. The combination of Horner and AD reactions was successfully used on one of stages of synthesis of the diterpenoid tonantzitlolone ( **276**) [162] (Scheme 90).

**Scheme 91:**
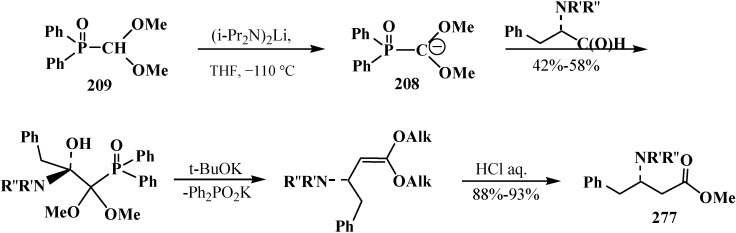
Synthesis of chiral β-amino acids **277** by the Horner reaction.

The involvement of chiral α-alkylaminoaldehydes in the Horner reaction, correspondingly, leads to the synthesis of esters of chiral β-amino acids **277** [20] (Scheme 91).

**Scheme 92:**
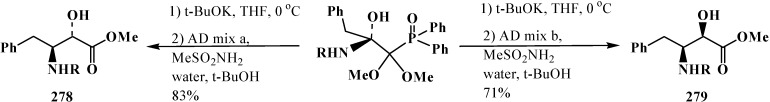
Using of combination of Horner and AD reactions to form α-hydroxy-β-amino acid diastereomers **278**, **279** (R = *t*-BuOC(O)).

Sharpless asymmetric dihydroxylation followed of Horner reaction also provides an opportunity to synthesize α-hydroxy-β-amino acid diastereomers **278**, **279** [20,128] (Scheme 92).

## 4. Phosphorylated Formaldehyde Halogenoaminals (Phosphorylated Vilsmeier–Haak Reagents) 17

**Figure 6:**
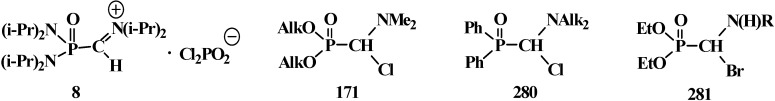
Representatives of phosphorylated Vilsmeier–Haak reagents **8**, **171**, **280**, **281** synthesized to date.

At present, four types of phosphorus compounds are known that can be related to phosphorylated Vilsmeier–Haak reagents: *N,N,N',N'*-tetraisopropyl [(*N'',N''*-diisopropylamino)methylydeniminium] phosphondiamide dichlorophosphate (**8**) [12], dialkyl [(*N,N*-dimethylamino)chloromethyl]phosphonates **171** [31,32], diphenyl[(*N,N*-dialkylamino)chloromethyl]phosphine oxides **280** [163], and structurally related to the phosphorylated Vilsmeier–Haak reagents, diethyl [(*N*-acylamino)bromomethyl]phosphonates **281** [164] (Figure 6).

No general methods of synthesis have been found for the compounds of this type **8**, **171**, **280**, **281**. Their chemical properties also differ significantly from each other.

### 4.1. Synthesis of N,N,N',N'-Tetraisopropyl [(N'',N''-Diisopropylamino)methylydeniminium] Phosphondiamide Dichlorophosphate (**8**)

**Scheme 93:**
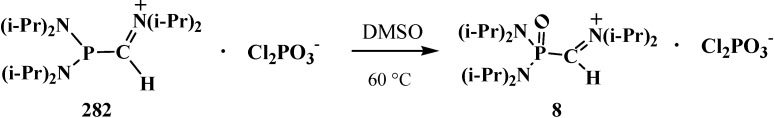
Phosphindiamide dichlorophosphate **282** oxidation by means of DMSO leads to the formation of phosphondiamide dichlorophosphate **8**.

A sole paper was published in 1999 [12] on the synthesis of *N,N,N',N'*-tetraisopropyl [(*N'',N''*-diisopropylamino)methylydeniminium]phosphondiamide dichlorophosphate (**8**) by the oxidation of *N,N,N',N'*-tetraisopropyl [(*N'',N''*-diisopropylamino)methylydeniminium]phosphindiamide dichlorophosphate (**282**) with dimethyl sulfoxide DMSO (Scheme 93). However, the chemical properties of **8** are insufficiently studied (for some chemical properties of **8** and its derivatives, see Section 1. Introduction).

### 4.2. Synthesis and Chemical Properties of Dialkyl [(N,N-Dimethylamino)chloromethyl]phosphonates **171**

The first compounds of this type, dimethyl [(*N,N*-dimethylamino)chloromethyl] phosphonate (**172**) and diethyl [(*N,N*-dimethylamino)chloromethyl]phosphonate (**173**) were synthesized in 1969 [31,32]. For the first time, these compounds were obtained as intermediates in the synthesis of tetraalkyl (*N,N*-dimethylaminomethyl)diphosphonates **174** by reaction of N,N-dimethyl(chloromethylideneiminium) chloride (**171**) with trialkyl phosphites **58** [32]—see Scheme 49, section “Diphosphinoyl *N,N*-dialkylaminomethanes”.

**Scheme 94:**
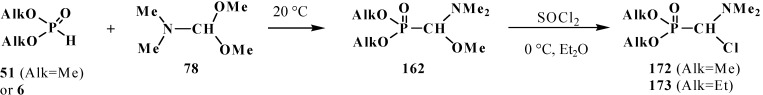
Reaction dialkyl phosphites **51** and dimethylformamide dimethylacetal (**78**) leads to phosphorilated halogenoaminals **172** (Alk = Me), **173** (Alk = Et).

Preparative amounts of these compounds were prepared by a two-step scheme [30,31]. First, the reaction of dimethylformamide dimethylacetal (**78**) with dialkyl phosphites **51** resulted in dialkyl [(*N,N*-dimethylamino)methoxymethyl]phosphonates **162**, which were further reacted with thionyl chloride (SOCl_2_) at 0 °C to yield the final products **172**, **173** (Scheme 94). See also Scheme 49 and Scheme 50.

**Scheme 95:**
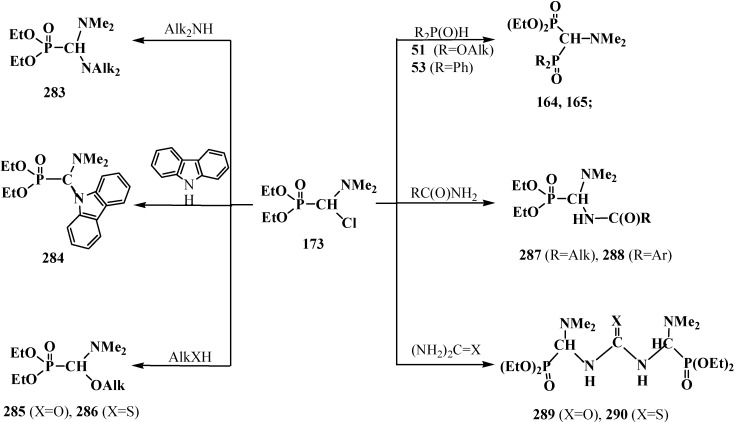
Reactions of diethyl [(*N,N*-dimethylamino)chloromethyl]phosphonate (**173**) with nucleophilic co-reagents.

This scheme is currently used [33,165]; phosphorus trichloride can be used instead of thionyl chloride [83]. Dimethyl [(*N,N*-dimethylamino)chloromethyl]phosphonate (**172**) was found to undergo spontaneous dealkylation on storage [31], therefore only diethyl [(*N,N*-dimethylamino)chloromethyl]phosphonate (**173**) is used in organic synthesis. Phosphonate **173**, as a phosphorylated Vilsmeier–Haak reagent where one chlorine atom is substituted by a diethoxyphosphoryl group, show electrophilic properties [166] inherent in compounds of such kind and readily reacts with nucleophiles. Thus, compound **173** reacts vigorously with secondary amines, carbazole, alcohols, thiols, and hydrophosphinoyl compounds **51**, **53** in the presence of equimolar amount of triethylamine. The reaction of α-phosphono-α-aminomethylation leads to the corresponding diethoxyphosphinoylformaldehyde derivatives: asymmetric aminals **283**, **284**, aminoacetals **285**, aminothioacetals **286**, and diphosphorylated *N,N*-dimethylaminomethanes **164** [30], including unsymmetrical **165** [165] (Scheme 95). The reaction with aromatic and aliphatic amides RC(O)NH_2_, urea and thiourea (NH_2_)_2_C=X [30] proceeds in a similar manner. Products of reactions are phosphorylated amidoaminals **287**, **288** and symmetrically disubstituted derivatives of urea and thiourea **289**, **290** (Scheme 95). See also Scheme 53, section “Diphosphinoyl *N,N*-dialkylaminomethanes”.

**Scheme 96:**
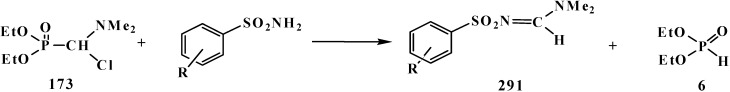
Cleavage of phosphorus–carbon bond by means of the reaction of compound **173** with arylsulfonamides.

Diethyl [(N,N-dimethylamino)chloromethyl]phosphonate (**173**) reacts with arylsulfonamides to lead to the cleavage of the phosphorus–carbon bond and give *N*-sulfonyl-substituted derivatives of formamidines **291** and diethyl phosphite (**6**) [30] (Scheme 96). See also Section 3.1. Cleavage of phosphorus–carbon bond.

**Scheme 97:**
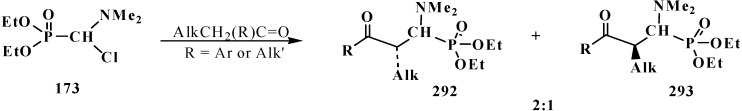
Reaction of compound **173** with ketones leads to diethyl [(*N,N*-dimethylamino)(aroyl(or alkanoyl)alkylmethyl)methyl]phosphonates **292** and **293**.

Compound **173** reacts with methyl aryl ketones [30] and dialkyl or aryl alkyl ketones [33] to give diethyl [(*N,N*-dimethylamino)(aroyl(or alkanoyl)alkylmethyl)methyl]phosphonates **292** and **293** [30,33], phosphorylated analogs of natural α-amino acids potentially possessing biological activity [33] (Scheme 97). However, reaction stereoselectivity is low, and the *anti*/*syn* ratio is 2:1.

**Scheme 98:**
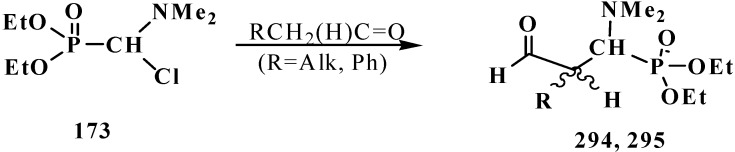
Reaction of compound **173** with aldehydes leads to phosphorylated aminoaldehydes **294** and **295**.

Like ketones, the reaction of **173** proceeds also with aldehydes branched at the α-position to the carbonyl group to give phosphorylated aminoaldehydes **294** and **295** (Scheme 98), but the reaction is not stereoselective: the stereoisomer ratio of the resulting α-formyl-α-methylaminophosphonates is 1:1 [33].

**Scheme 99:**
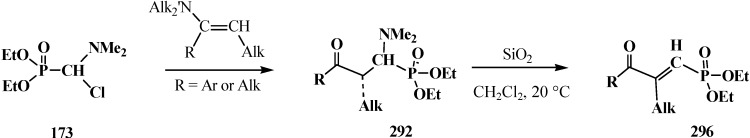
Synthesis of diethyl (α-methylamino-α-aroyl(or alkanoyl))methylphosphonates **292** and phosphorylated vinyl ketones **296**.

The involvement of enamines instead of ketones as their synthetic equivalents in the reaction with phosphonate **173** provides stereoselective synthesis of diethyl (α-methylamino-α-aroyl(or alkanoyl))methylphosphonates **292**; the reaction leads to the products with only *anti* configuration [33] (Scheme 99).

Diethyl [(*N,N*-dimethylamino)(aroyl(or alkanoyl)alkylmethyl)methyl]phosphonates **292**, **293** (Scheme 101, Scheme 102 and Scheme 103) are obtained as hydrochlorides in the above-stated syntheses. They are unstable and on storage undergo spontaneous β-elimination of dimethylamino group as dimethylammonium chloride—with conversion up to 40% over 24 h [30] to form the corresponding phosphorylated vinyl ketones **296**. The same result was achieved on the heating of hydrochlorides of compounds **292** and **293** to 100 °C over 45 min [30] or stirring their solutions in methylene chloride with silica gel at 20 °C for 15 h [33]. The reaction of amino group elimination with the use of silica gel is also stereoselective: the anti stereoisomers **292** produce only *E* isomers of vinyl compounds **297** [33] (Scheme 99).

**Scheme 100:**
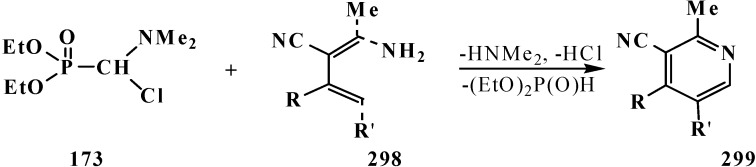
The reaction of compound **173** with β-enaminonitriles **298** leads to nitriles of nicotinic acid **299**.

The reaction of β-enaminonitriles **298** with compound **173** was used in the synthesis of substituted nitriles of nicotinic acid **299** [167] (Scheme 100).

**Scheme 101:**
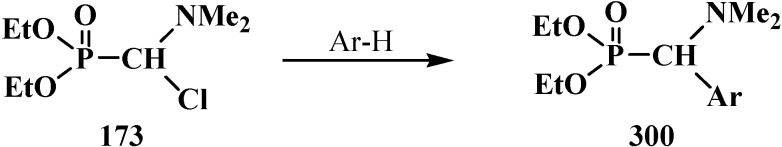
Interactions of phosphonate **173** with aromatic compounds (Ar = 4-dimethylaminophenyl; 2-hydroxynaphthyl-1; 2-hydroxy(5-methyl)phenyl; 1-methylaminoindol-3-yl; 1,5-dimethylaminopyrrol-2-yl; 5-methylfur-2-yl).

As a phosphorylated Vilsmeier–Haak reagent, phosphonate **173** undergoes typical reactions with activated aromatic compounds: *N,N*-dimethylaniline, triethylammonium salts of β-naphthol and *p*-cresol, and heteroaromatic compounds: *N*-methylindole, *N*-methylpyrrole, α-methylfuran. The synthesis of aromatic α-aminomethylphosphonates **300** is regiospecific: only one addition product forms in each case [33] (Scheme 101).

### 4.3. Syntheses and Chemical Properties of Diphenyl[(N,N-dialkylamino)chloromethyl]phosphine Oxides **280**

**Scheme 102:**
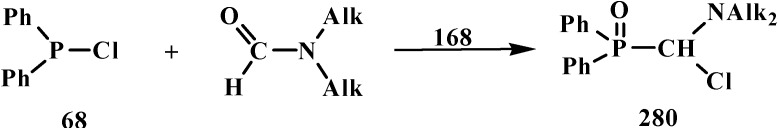
Synthesis of diphenyl[(*N,N*-dialkylamino)chloromethyl]phosphine oxides **280**.

Diphenyl[(*N,N*-dialkylamino)chloromethyl]phosphine oxides **280** are the diphenylphosphinoyl analogs of dialkyl [((*N,N*-dialkylamino)chloromethyl]phosphonates. They are obtained by the reaction of chlorodiphenylphosphine (**68**) with *N,N*-dialkylformamides in the presence of 10%–20% of *N,N*-dialkylchloromethyleniminium chloride [Alk_2_N=C(H)Cl]^+^Cl^−^ (**168**)—Vilsmeier–Haak reagent [168,169] according to Scheme 102.

**Scheme 103:**
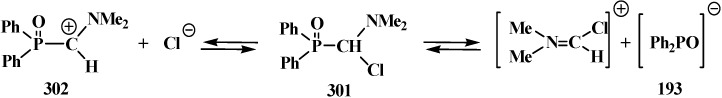
Diphenyl[(*N,N*-dimethylamino)chloromethyl]phosphine oxide (**301**) dissociation in solutions with a cleavage as bond **C-Cl** and bond **C-P**.

Although phosphine oxides **280** are the formal analogs of dialkyl [(*N,N*-dialkylamino)chloromethyl]phosphonates **171**, they differ substantially in reactivity. It was shown by the example of diphenyl[(*N,N*-dimethylamino)chloromethyl]phosphine oxide (**301**) that compounds **280** can dissociate in solutions with the cleavage of C–Cl and C–P bonds. See also section “Cleavage of phosphorus–carbon bond”. The dissociation in solution results in the formation of both diphenyl[(*N,N*-dimethylamino)chloromethyliden]phosphine oxide cation (**302**) and diphenylphosphinite anion (**193**) [170] (Scheme 103).

**Scheme 104:**
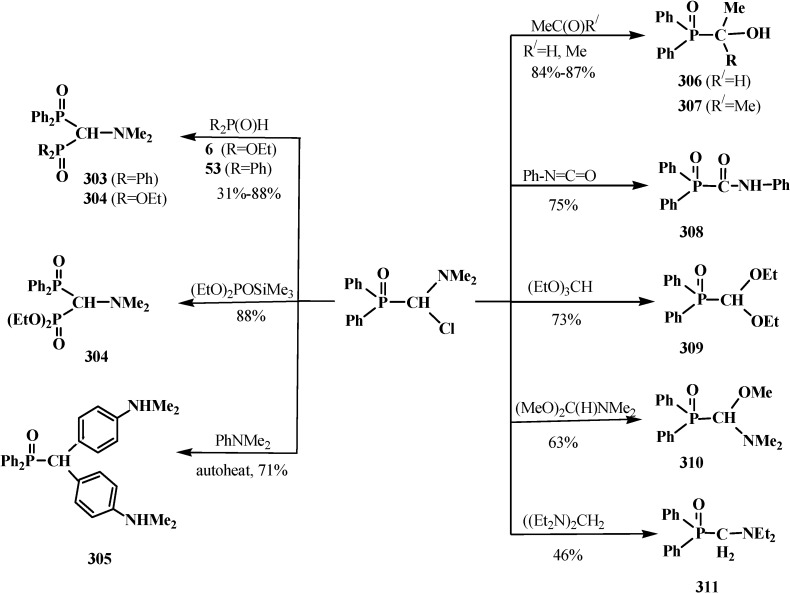
Reactions of diphenyl[(*N,N*-dimethylamino)chloromethyl]phosphine oxide (**280**) with nucleophilic and electrophilic coreagents.

Therefore there are two kinds of reactivity for **301** (and accordingly **280**) [163,170]: electrophilic diphenyl[(*N,N-*dimethylamino)chloromethylidene]phosphine oxide cation (**302**) reacts with nucleophiles such as hydrophosphinoyl compounds **6**, **53**, mixed diethyl trimethylsilyl phosphite (EtO)_2_POSiMe_3_ and *N,N*-dimethylamino aniline PhNMe_2_, to give symmetrical **303**, R=Ph and unsymmetrical **304**, R=OEt diphosphorylated *N,N*-dimethylaminomethanes and diphenyl[(bis(4-*N,N*-dimethylamino)phenyl)methyl] phosphine oxide (**305**), the product of substitution of both chloro and amino groups at the carbon atom of **301**. However, nucleophilic diphenylphosphinite anion (**193**) reacts with electrophiles: acetic aldehyde MeC(O)H, acetone Me_2_C(O), phenyl isocyanate PhNCO, triethyl orthoformate (EtO)_3_CH, dimethylformamide dimethylacetal (**79**), and bis(diethylamino)methane (Et_2_N)_2_CH_2_ to form addition or substitution products, α-phosphorylated compounds: alcohols **306** and **307**, carbamoyl compound **308**, acetal **309**, aminoacetal **310** and amine **311** (Scheme 104).

### 4.4. Synthesis and Chemical Properties of Diethyl [(N-Acylamino)bromomethyl]phosphonates **281**

**Scheme 105:**
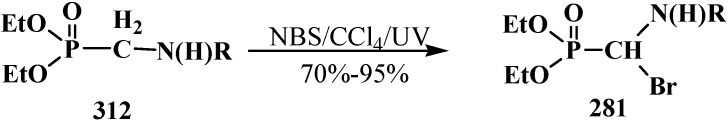
Synthesis of diethyl [(*N*-acylamino)bromomethyl]phosphonates **281** where R = AlkC(O), ArC(O), AlkS(O)_2_, ArS(O)_2_.

Diethyl [(*N*-acylamino)bromomethyl]phosphonates **281** were prepared in high yields by the bromination of diethyl (*N*-acylamino)methylphosphonates **312** with N-bromosuccinimide (NBS) in carbon tetrachloride [164,171] (Scheme 105).

**Scheme 106:**
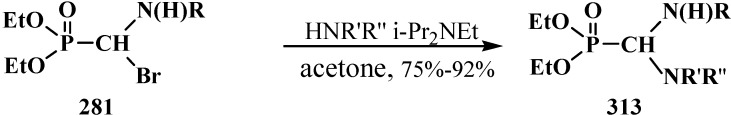
Transformation of bromomethylphosphonates **281** into unsymmetrical amidoaminals **313**, where HNR'R'' are morpholine, piperidine, pyrrole, 2-aminomethyltetrahydrofuran, *N*-methylbenzylamine, and aniline.

To date diethyl [(*N*-acylamino)bromomethyl]phosphonates **281** remain poorly studied, and their chemical properties are insufficiently studied. Nonetheless, compounds **281**, structurally related to the phosphorylated Vilsmeier–Haak reagents, behave as electrophiles similarly to dialkyl [(*N,N*-dimethylamino)chloromethyl]phosphonates **171** [166]. They react readily with such nucleophiles as primary aliphatic and aromatic amines and secondary cyclic amines in the presence of ethyldiisopropylamine—Hünig’s base as well as triethylamine (Scheme 106). Reaction products are diethyl [(*N*-acylamino)aminomethyl]phosphonates **313**, unsymmetrical amidoaminals of diethyl (formyl)phosphonate (**3**) [30] showing biological and pharmacological activity [172].

**Scheme 107:**
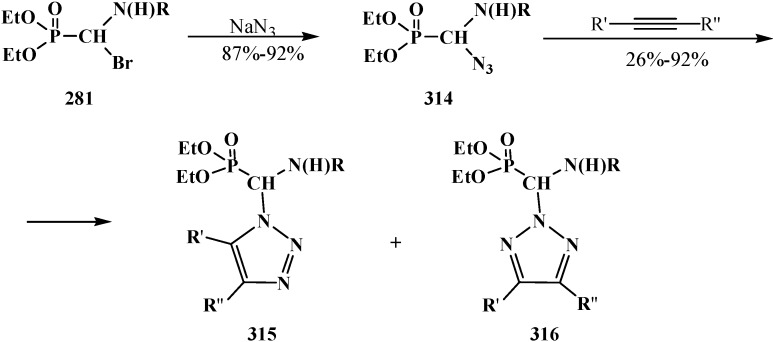
Synthesis of (triazolylmethyl)phosphonates **315** and **316** from bromomethylphosphonates **281**, where R', R'' = AlkC(O), ArC(O), AlkS(O)_2_, ArS(O)_2_.

The reaction of compounds **282** with sodium azide leads to diethyl [(*N*-acylamino)azidomethyl]-phosphonates **314**, valuable precursors in the synthesis of phosphinoyl-substituted 1,2,3-triazoles, the products of 1,3-dipolar addition to disubstituted alkynes [173,174]. A mixture of the resultant two regioisomers of diethyl [(*N*-acylamino)(1-(1,2,3-triazolyl))methyl]phosphonates (**315**) and diethyl [(*N*-acylamino)(2-(1,2,3-triazolyl))methyl]phosphonates (**316**) can be separated by chromatography on silica gel (Scheme 107).

**Scheme 108:**
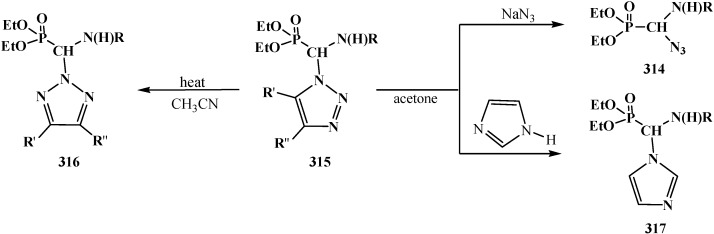
Reaction replacement of 1,2,3-triazole group of compound **315** with nucleophiles to give compounds **314** and **317** and isomerisation of **315** into compound **316**.

Prolonged heating of acetonitrile solutions of **315** leads to migration of [(*N*-acylamino)(diethylphosphinoyl)]methyl group from the first to the second nitrogen atom (N1–N2 migration) of 1,2,3-triazole group to give **316**. In the presence of nucleophiles, azide ion or imidazole, the triazole fragment is displaced to yield diethyl [(*N*-acylamino)azidomethyl]phosphonates **314** or diethyl [(*N*-acylamino)(1-imidazolyl)methyl]phosphonates **317** (Scheme 108) [174].

**Scheme 109:**
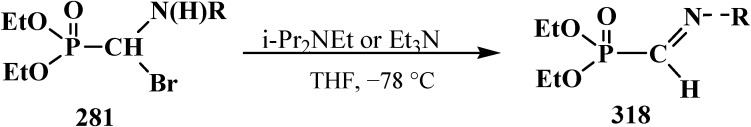
Synthesis of diethyl [(*N*-acyl)iminomethyl]phosphonates (**318**) from phosphonates **281**.

The treatment of phosphonates **281** with Hünig’s base i-Pr_2_NEt [164] or triethylamine Et_3_N [171] in tetrahydrofuran at −78 °C was shown to result in diethyl [(N-acyl)iminomethyl]phosphonates **318** in yields up to 98% (Scheme 109).

## 5. Alkyl (dialkoxymethyl)phosphinates—H-Phosphinates 34. Syntheses and Chemical Properties

**Scheme 110:**
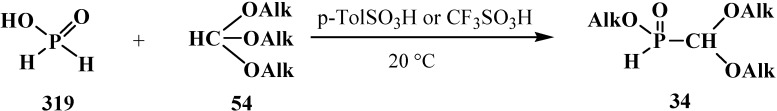
Synthesis of alkyl (dialkoxymethyl)phosphinates **34**.

The synthesis of alkyl (dialkoxymethyl)phosphinates—H-phosphinates **34** in yields up to 96% by the reaction of hypophosphorous acid (**319**) with orthoformates **54** in the presence of catalytic amounts amounts of *p*-toluenesulfonic acid (p-TolSO_3_H) [57,175] or trifluoroacetic acid (CF_3_SO_3_H) [58] was reported in 1977 (Scheme 110). The method is still used at present.

The chemical properties of phosphinates **34** were studied almost exclusively by the example of ethyl (diethoxymethyl)phosphinate (**86**). Ethyl (diethoxymethyl)phosphinate (**86**) retains properties typical for both phosphorus esters and hydrophosphoryl compounds and retains the general ability of phosphorylated formaldehyde acetals to undergo the cleavage of the phosphorus–carbon bond.

**Scheme 111:**
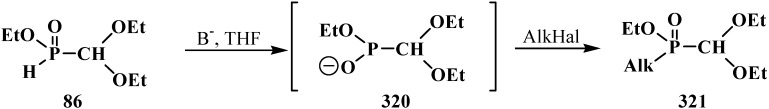
Phosphinate **86** alkylation with alkylhalydes.

Phosphinate **86** is readily alkylated under the action of alkyl halides in the presence of bases: Na, NaH, BuLi (B^−^) (via anion **320**) to give ethyl alkyl(diethoxymethyl)phosphinates **321** [58,176] (Scheme 111).

**Scheme 112:**
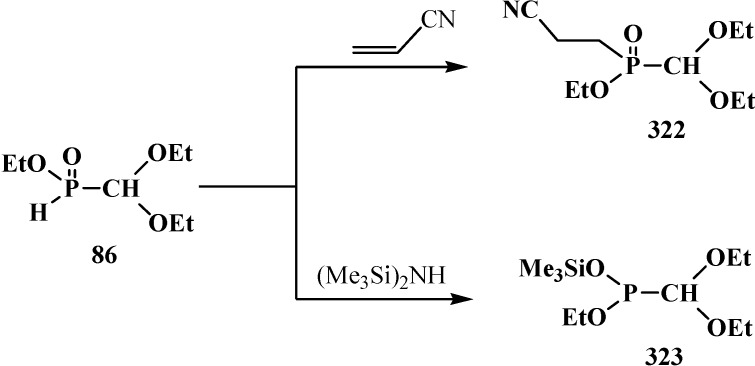
Transformations of compound **86** into phosphinate **322** and a three-coordinated phosphorus compound (phosphinite **323)**.

Compound **86** also readily undergoes addition to activated double bonds, or it can transform into three-coordinated phosphorus compounds [59,176,177], for example, in Scheme 112, compounds **322** and ethyl trimethylsilyl (diethoxymethyl)phosphinite (**323**), respectively.

**Scheme 113:**
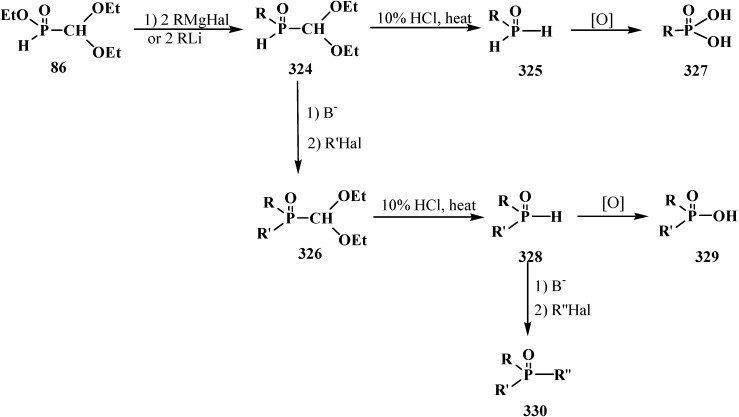
Using phosphinate **86** for the synthesis of phosphine oxides **325**, **328**, **330**, phosphonic **328** and unsymmetrical phosphinic acids **329**.

It also undergoes Todd–Atherton reaction with phenols and cross-coupling reactions with aryl bromides [106] in the presence of tetrakis(triphenylphosphine)palladium(0), Pd(Ph_3_P)_4_, see Scheme 17. In **86**, one of two P–H bonds of initial hypophosphorous acid **319** is protected by a diethoxymethyl group and can be restored in subsequent stages after deprotection [58,176]. When treated with two equivalents of organomagnesium or organolithium compounds, phosphinate **86** produces secondary phosphine oxides **324** [178], a hidden form of unstable primary phosphine oxides **325** apt to disproportionation [179], that can be easily obtained by subsequent acid hydrolysis. Further alkylation of secondary phosphine oxides **324** in the presence of a base leads to unsymmetrical tertiary phosphine oxides **326**, which in turn are the hidden form of unsymmetrical secondary phosphine oxides **328**. Similarly to primary phosphine oxides **325**, compounds **327** can be also obtained by subsequent acid hydrolysis of tertiary phosphine oxides **326**. Further oxidation leads to the corresponding phosphonic **328** and unsymmetrical phosphinic acids **329**. Unsymmetrical tertiary phosphine oxides **330** can be obtained by the following alkylation of secondary phosphine oxides **327** (Scheme 113).

**Scheme 114:**
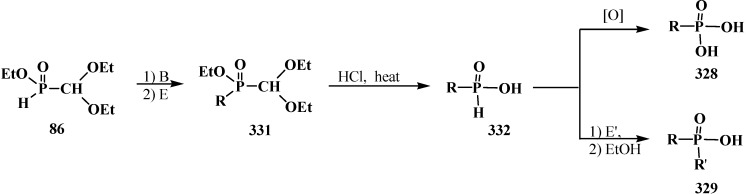
Phosphonic **328**, H-phosphinic **332** and unsymmetrical phosphinic acids **329** syntheses starting with the reaction of **86** with electrophiles, where E = RHal or olefins with the activated double bond.

Phosphonic and unsymmetrical phosphinic acids **328** and **329** can also be obtained by the scheme that begins with the alkylation reaction by introducing **86** in the reaction of one or sequentially both P–H bonds, thus solving the problem of selectivity [58,59,176,178], over phosphinates **331** and H-phosphinic acids **332**, respectively. The scheme allows one to avoid the use of highly toxic hypophosphorous acid in the syntheses [177] (Scheme 114).

**Scheme 115:**
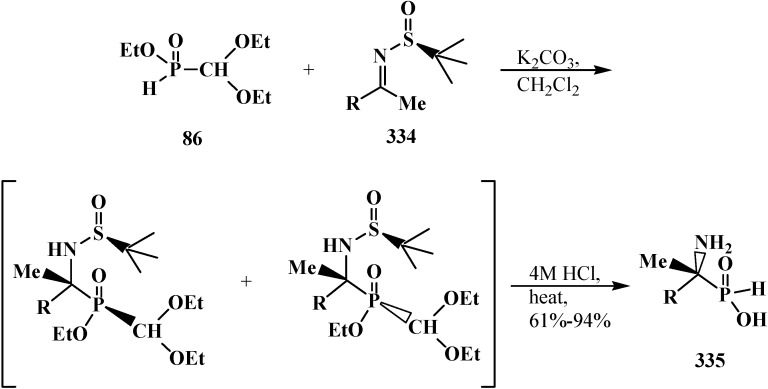
Synthesis of H-phosphinic analogs of natural α-amino acids **333** by means of the reaction of phosphinate **86** with chiral (*S*)-*N*-*tert*-butylsulfinylketimines **334**.

Due to the unique combination of chemical properties, phosphinate **86** is used in modern organic synthesis and synthesis of biologically active compounds. H-phosphinic analogs of natural α-amino acids **333** were obtained with >95% enantiomeric excess by the reaction of **86** with chiral (*S*)-*N*-*tert*-butylsulfinylketimines (**334**) [180] (Scheme 115).

**Scheme 116:**
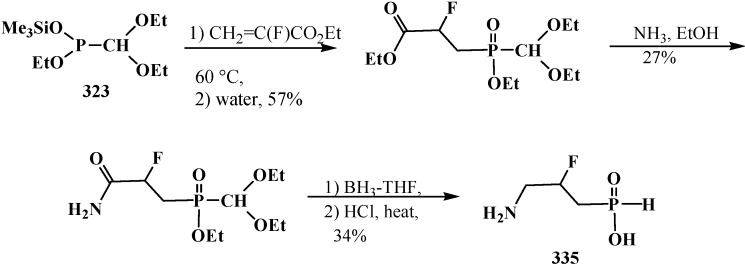
Silylated compound **323** used for the synthesis of γ-aminobutyric acid analog **335**.

3-Amino-2-fluoropropyl-*H*-phosphinic acid (**335**), a γ-aminobutyric acid (GABA) analog, a potential pharmaceutical for the treatment of central nervous system diseases, was prepared from compound **323** as the silylated form of ethyl (diethoxymethyl)phosphinate **86** [181] (Scheme 116).

**Scheme 117:**
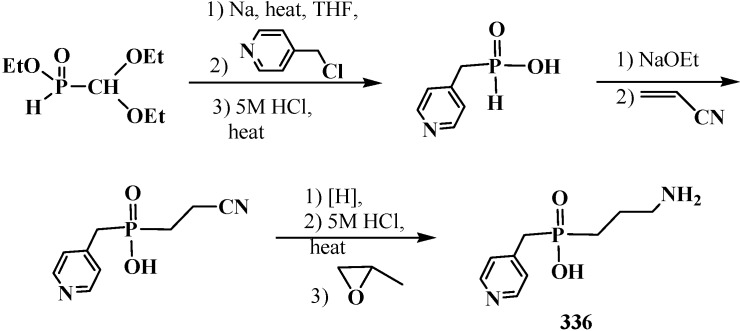
Synthesis of potential neurotropic pharmaceutical, GABA antagonist **336**.

Disubstituted 3-aminopropyl-4-pyridylphosphinic acid (**336**), a potential neurotropic pharmaceutical and GABA antagonist, was obtained in a similar manner [182] (Scheme 117).

## 6. Phosphorylated Formaldehyde Hydrates—Geminal Diols 35. Syntheses and Chemical Properties

**Scheme 118:**
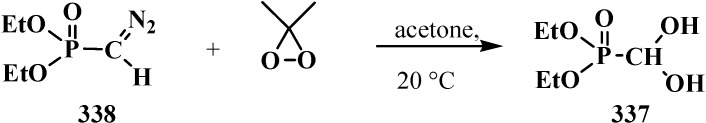
Synthesis of diethyl (dihydroxymethyl)phosphonate (**337**) by the reaction of diazomethylphosphonate (**338**) with 3,3-dimethyldioxirane.

The chemistry of phosphorylated formaldehyde hydrates **35** began to develop since the mid 1990s when diethyl (dihydroxymethyl)phosphonate (**337**) was obtained in quantitative yield by the reaction of diethyl (diazomethyl)phosphonate (**338**) with 3,3-dimethyldioxirane—acetone peroxide at 20 °C [60,61] (Scheme 118).

**Scheme 119:**
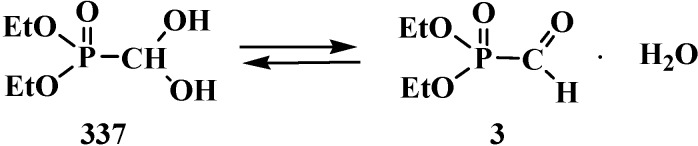
Equilibrium between diethyl (dihydroxymethyl)phosphonate (**337**) and the hydrated form of diethyl formylphosphonate **3**.

It was shown that **337** exists in an equilibrium with the hydrated form of diethyl formylphosphonate **3** [60,61] (Scheme 119).

The chemical properties of phosphonate **337** provide the possibility of considering it as a hidden form of phosphorylated formaldehyde. Compound **337** reacts with secondary amines R'_2_NH with the cleavage of the phosphorus–carbon bond and formation of the corresponding *N,N*-disubstituted formamides and diethyl phosphite (**6**) [60,61] (it behaves as a source of formyl group [C(O)H]^+^ cation, synthon type a^1^ [183,184].

**Scheme 120:**
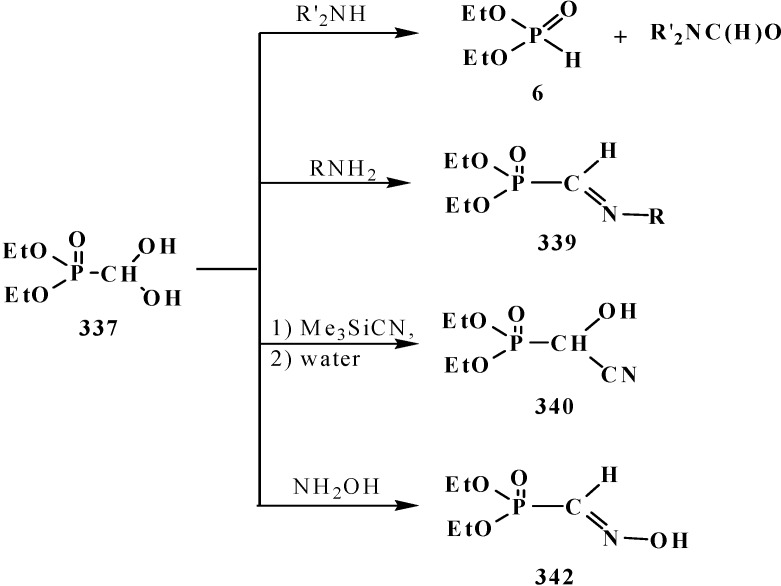
Reactions of compound **337** with some coreagents, where R= *t*-Bu or Ph.

Compound **337** also shows properties of a typical aldehyde when reacted with primary amines RNH_2_, trimethylsilyl cyanide Me_3_SiCN, and hydroxylamine NH_2_OH to give phosphorylated formaldimines **339**, formalcyanohydrin **340**, and formaldoxime **341**, respectively [60,61] (Scheme 120).

**Scheme 121:**
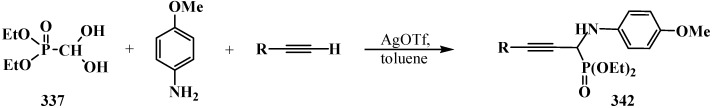
Catalytical synthesis of α-aminopropargylphosphonates **342** by a Mannich reaction.

As a hidden form of diethyl (formyl)phosphonate (**3**), phosphonate **337** in the presence of silver trifluoroacetate AgOTf as a catalyst undergoes a Mannich reaction with anisidine and terminal alkyl- or aryl-substituted alkynes. Reaction products, α-aminopropargylphosphonates **342**, are valuable precursors in the synthesis of α-aminophosphonic acids showing high biological activity as α-amino acid mimetics [63] (Scheme 121).

**Scheme 122:**
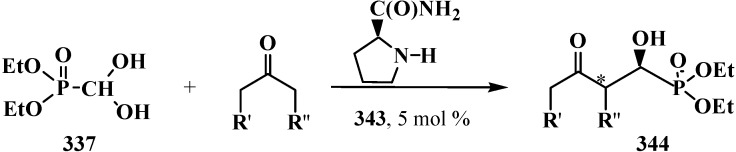
Synthesis of diastereomeric α-hydroxyphosphonates **344** by asymmetrical cross-aldol condensation.

In the presence l-proline amide (**343**) as a catalyst, **337** undergoes an asymmetrical cross aldol condensation with aliphatic ketones. Diastereomerically pure α-hydroxyphosphonates **344**, precursors of α-hydroxyphosphonic acids, result from chirality induction with diastereomeric excesses greater that 99% [185] (Scheme 122).

Like α-aminophosphonic acids, α-hydroxyphosphonic acids are also α-amino acid mimetics and exhibit high biological activity [185]. Similar results were obtained in the aldol condensation of ketones with racemic ethyl phenyl(dihydroxymethyl)phosphinate (**345**). In this case, the final products are α-hydroxyphosphinic acids **346** [62].

## 7. Conclusions

Phosphorylated formaldehyde derivatives, *i.e.*, acetals and related compounds, are a group of largely underinvestigated species. The experimental data accumulated since the early 1960s confirm that these compounds can be used in a wide variety of syntheses that have not been fully realized to date. For this reason, the growth of interest to these compounds will allow investigating their chemical properties in more detail and potentially enrich organic and organoelement chemistry with new synthetic methods.
